# Medusavirus, a Novel Large DNA Virus Discovered from Hot Spring Water

**DOI:** 10.1128/JVI.02130-18

**Published:** 2019-04-03

**Authors:** Genki Yoshikawa, Romain Blanc-Mathieu, Chihong Song, Yoko Kayama, Tomohiro Mochizuki, Kazuyoshi Murata, Hiroyuki Ogata, Masaharu Takemura

**Affiliations:** a Institute for Chemical Research, Kyoto University, Gokasho, Uji, Kyoto, Japan; b National Institute for Physiological Sciences, Okazaki, Aichi, Japan; c Earth-Life Science Institute, Tokyo Institute of Technology, Meguro, Tokyo, Japan; d Faculty of Science, Tokyo University of Science, Shinjuku, Tokyo, Japan; University of Illinois at Urbana Champaign

**Keywords:** NCLDV, *Acanthamoeba*, cryo-electron microscopy, genome sequencing, giant virus, histone, lateral gene transfer, major capsid protein, molecular phylogenetic analysis

## Abstract

Recent discoveries of new large DNA viruses reveal high diversity in their morphologies, genetic repertoires, and replication strategies. Here, we report the novel features of medusavirus, a large DNA virus newly isolated from hot spring water in Japan. Medusavirus, with a diameter of 260 nm, shows a T=277 icosahedral capsid with unique spherical-headed spikes on its surface. It has a 381-kb genome encoding 461 putative proteins, 86 of which have their closest homologs in Acanthamoeba, whereas 279 (61%) are orphan genes. The virus lacks the genes encoding DNA topoisomerase II and RNA polymerase, showing that DNA replication takes place in the host nucleus, whereas the progeny virions are assembled in the cytoplasm. Furthermore, the medusavirus genome harbored genes for all five types of histones (H1, H2A, H2B, H3, and H4) and one DNA polymerase, which are phylogenetically placed at the root of the eukaryotic clades. In contrast, the host amoeba encoded many medusavirus homologs, including the major capsid protein. These facts strongly suggested that amoebae are indeed the most promising natural hosts of medusavirus, and that lateral gene transfers have taken place repeatedly and bidirectionally between the virus and its host since the early stage of their coevolution. Medusavirus reflects the traces of direct evolutionary interactions between the virus and eukaryotic hosts, which may be caused by sharing the DNA replication compartment and by evolutionarily long lasting virus-host relationships. Based on its unique morphological characteristics and phylogenomic relationships with other known large DNA viruses, we propose that medusavirus represents a new family, Medusaviridae.

**IMPORTANCE** We have isolated a new nucleocytoplasmic large DNA virus (NCLDV) from hot spring water in Japan, named medusavirus. This new NCLDV is phylogenetically placed at the root of the eukaryotic clades based on the phylogenies of several key genes, including that encoding DNA polymerase, and its genome surprisingly encodes the full set of histone homologs. Furthermore, its laboratory host, Acanthamoeba castellanii, encodes many medusavirus homologs in its genome, including the major capsid protein, suggesting that the amoeba is the genuine natural host from ancient times of this newly described virus and that lateral gene transfers have repeatedly occurred between the virus and amoeba. These results suggest that medusavirus is a unique NCLDV preserving ancient footprints of evolutionary interactions with its hosts, thus providing clues to elucidate the evolution of NCLDVs, eukaryotes, and virus-host interaction. Based on the dissimilarities with other known NCLDVs, we propose that medusavirus represents a new viral family, Medusaviridae.

## Abstract (precis)

We have isolated a new nucleocytoplasmic large DNA virus (NCLDV) from hot spring water in Japan, named medusavirus. This new NCLDV is phylogenetically placed at the root of the eukaryotic clades based on the phylogenies of several key genes, including that encoding DNA polymerase, and its genome surprisingly encodes the full set of histone homologs. Furthermore, its laboratory host, Acanthamoeba castellanii, encodes many medusavirus homologs in its genome, including the major capsid protein, suggesting that the amoeba is the genuine natural host from ancient times of this newly described virus and that lateral gene transfers have repeatedly occurred between the virus and amoeba. These results suggest that medusavirus is a unique NCLDV preserving ancient footprints of evolutionary interactions with its hosts, thus providing clues to elucidate the evolution of NCLDVs, eukaryotes, and virus-host interaction. Based on the dissimilarities with other known NCLDVs, we propose that medusavirus represents a new viral family, Medusaviridae.

## INTRODUCTION

Viruses infect all living things from three domains of life (Bacteria, Archaea, and Eukarya), and they represent the most abundant and ubiquitous biological entities on the planet (1–3). As mandatory intracellular parasites, viruses evolve in close physical contact with their hosts and drive the evolution of the host via gene transfer (4–6) and coevolutionary arms races (7–10). The major virological discoveries of the last few decades are the successive discoveries of the diverse nature of the virosphere with respect to virion structures (3, 11–13), genetic repertoire (14–16), and various replication strategies in the cell (17–19). Despite the puzzling origin and diversity of viruses, recent structural and phylogenomic analyses yielded evidence suggesting that viruses emerged in the very early stages of evolution (20–23), which may account for the enormous diversity of modern viruses (24).

Nucleocytoplasmic large DNA viruses (NCLDVs) form a monophyletic group of eukaryotic viruses with large and complex double-stranded DNA (dsDNA) genomes ranging from 100 kbp to 2.50 Mbp (25, 26). The NCLDVs originally encompassed the families Poxviridae, Asfarviridae, Iridoviridae, Ascoviridae, and Phycodnaviridae (27), and have subsequently expanded with the discoveries of new groups of giant viruses infecting amoebae, including Mimiviridae (28), Marseilleviridae (29), pandoraviruses (14), pithoviruses (30), faustoviruses (31), mollivirus (32), kaumoebavirus (33), cedratviruses (34), and pacmanvirus (35). Their virions display substantial morphological variations. Many of the NCLDVs present large icosahedral capsids composed of double-jelly roll major capsid proteins (MCPs), but exceptions to this virion architecture are seen in Poxviridae, with brick- or ovoid-shaped virions; Ascoviridae, with bacilliform or allantoid capsids; mollivirus, with a spherical virion; and pandoraviruses and pithoviruses, with amphora-shaped virions (11, 36). The host organisms of NCLDVs span a wide range of eukaryotes, namely Unikonts (Metazoa and Amoebozoa), Plantae (green algae), and Chromalveolata (Haptophyta, Alveolata, and stramenopiles) (36). Some NCLDVs replicate exclusively in the cytoplasm, whereas others replicate using both nuclear and cytoplasmic compartments (17).

NCLDVs and other large DNA viruses acquired many genes from the cellular genome during evolution (37, 38). However, phylogenetic analyses suggest that essential DNA processing proteins of eukaryotes, including DNA polymerases, originally derived from viruses (39–41). Phylogenies of some DNA repair enzymes (42, 43) and DNA-dependent RNA polymerases (44) of NCLDVs are also consistent with their early divergence, prior to radiation of the eukaryotic domain. Another notable feature of NCLDVs is that they encode many unique but functionally unknown genes, i.e., orphan genes (ORFans) or lineage-specific genes, which may be the result of ongoing *de novo* creation of genes in their genomes (45, 46). The ancient origin and unique features of these large DNA viruses prompted biologists to propose theories to interconnect viruses with major evolutionary transitions (47–49), such as the emergence of the DNA replication machinery (possibly including the DNA itself) (39, 50, 51) and the emergence of the eukaryotic nucleus (52, 53).

Amoebae are a potent tool to isolate novel large DNA viruses. Currently, viruses isolated using amoeba coculture are classified into at least nine groups, as mentioned above. All of these viruses belong to the NCLDVs, though this classification is controversial for some of the viruses (11, 54). However, the actual natural hosts of these viruses have not yet been determined. In the present study, we newly isolated “Acanthamoeba castellanii medusavirus (Medusavirus)” from a muddy freshwater sample spilled out from hot spring in Japan, using an amoeba coculture method. Some unique features of the newly identified virus assigned medusavirus to a new family of NCLDVs and led us to conclude that amoeba is indeed the most promising natural host and that lateral gene transfers (LGTs) have taken place repeatedly and bidirectionally between the virus and its host since the early stages of their coevolution.

## RESULTS

### Isolation of medusavirus.

**FIG 1:**
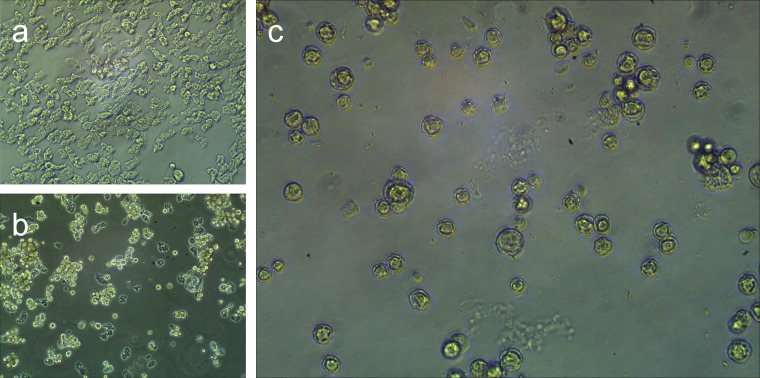
Phase-contrast micrographs of A. castellanii cells. (a) A. castellanii cells without viral infection. (b) A. castellanii cells 1 to 2 days PI. (c) Encystment of A. castellanii cells around 2 days PI.

Medusavirus was isolated using Acanthamoeba castellanii as the laboratory host. Cytopathic effects, such as cell rounding of host cells due to viral infection, were observed 1–2 days postinfection (PI) (Fig. 1a and b). Eventually, the viral infection induced amoebae to undergo morphological changes similar to encystment in a subpopulation of amoeba cells as early as 2 days PI (Fig. 1c). On the other hand, other amoeba cells without encystment were lysed. This encystment-like phenomenon prompted us to name this virus after Medusa, the monster in ancient Greek mythology who turns onlookers to stone.

### Unique particle morphology.

**FIG 2:**
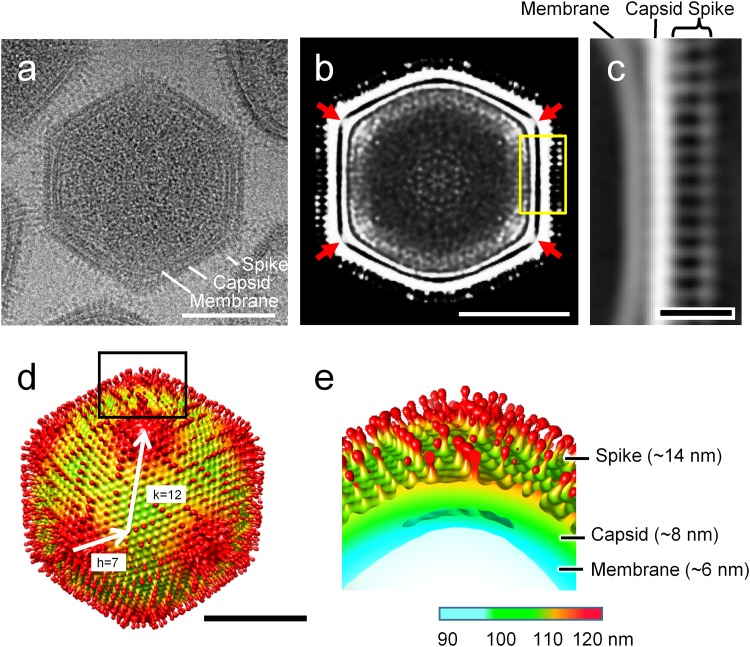
Structure of medusavirus. (a) Cryo-EM image of a DNA-filled medusavirus particle viewed from a 3-fold axis. Spike, capsid, and membrane are labeled. (b) Center slice of 3D reconstruction of the DNA-filled medusavirus particle viewed from the 2-fold axis. The internal membrane was extended and directly connected with the capsid under the 5-fold axes (red arrows). (c) Enlargement of the surface area of the medusavirus particle (yellow box in panel b). Spike, capsid, and membrane are labeled. (d) Surface view of 3D reconstruction of the medusavirus particle. The radiuses in the particle are indicated with the color map in panel e. (e) Extraction of the periphery of the 5-fold axis in panel d. Spike, capsid, and membrane are labeled with widths. Bars a, b, and d = 100 nm; scale bar c = 20 nm.

Single-particle cryo-electron microscopy (cryo-EM) analysis revealed that the medusavirus virion is an icosahedron with T=277 (h=7; k=12) and is approximately 260 nm in diameter (Fig. 2a and d). The single-layered major capsid (approximately 8 nm in thickness) of medusavirus was covered with a number of spherical-headed spikes of approximately 14 nm in length, each of which extended from each capsomer (Fig. 2). The virus particles isolated in the laboratory were either filled with DNA or lacked DNA inside. The resolutions of the cryo-EM maps were estimated at approximately 31.7 Å for the DNA-filled particle and approximately 31.3 Å for the empty particle. The spikes appeared to be rather flexible because they seemed to be blurred in the DNA-filled matured particle or the higher-resolution map with imposed icosahedral symmetry. The viral capsid was backed with a 6-nm thick internal membrane, as commonly found in NCLDVs (Fig. 2a, b, c and e). The membrane extended and directly interacted with the inner surface of the capsid under the 5-fold axis (red arrows in Fig. 2b).

### Entry of medusavirus DNA to the nucleus.

**FIG 3:**
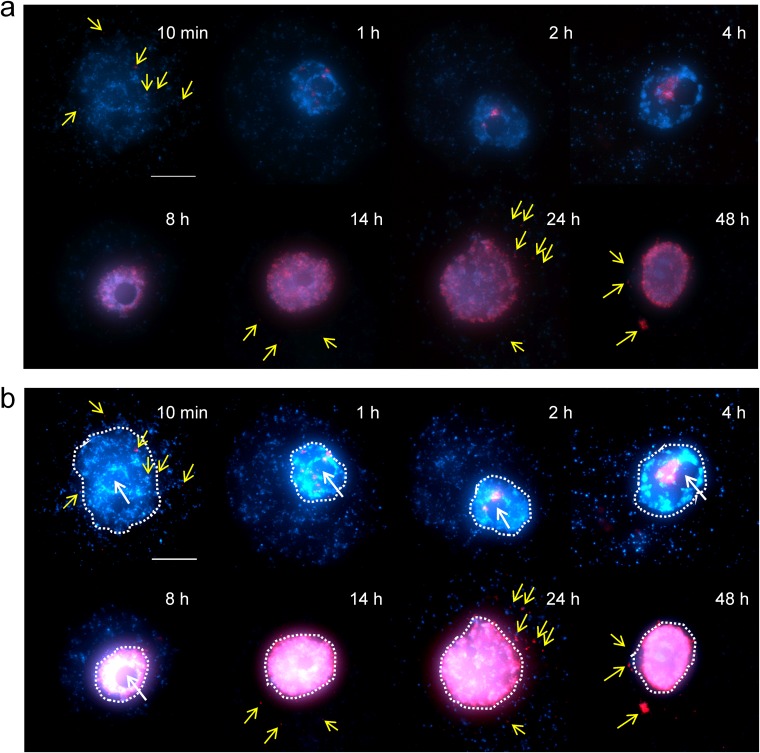
Time-dependent fluorescence *in situ* hybridization (FISH) analysis of medusavirus DNA localization in medusavirus-infected A. castellanii cells. Red, medusavirus DNA labeled by Cy-3; blue, both A. castellanii genomic DNA and medusavirus DNA stained by DAPI (4′,6-diamidino-2-phenylindole). Yellow arrows indicate medusavirus DNA signals in the cytoplasm of host cells. The sampling times after virus infection are indicated on the top right corner of each panel. Bar, 10 μm. (a) Original images. (b) Brighter images. Contrast was digitally enhanced. Dashed lines indicate host cell nucleus.

**FIG 4:**
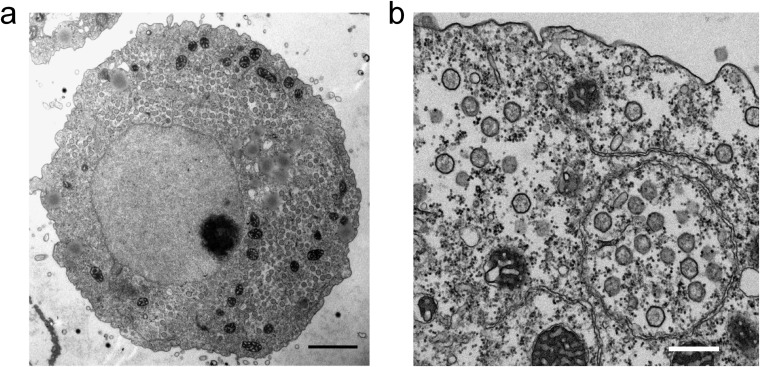
Electron micrographs of A. castellanii cell infected with medusavirus at 8 to 10 h PI. (a) Entire A. castellanii cell filled with freshly replicated medusavirus capsids. (b) A closeup view of the cytoplasm of A. castellanii cell filled with freshly replicated medusavirus capsids. Numerous capsids not filled with the viral DNA are observed. Bar a = 2 μm; bar b = 500 nm.

Fluorescent *in situ* hybridization (FISH) analysis revealed that medusavirus DNA was localized in the nucleus of amoeba cells by 1 h PI. Signals of medusavirus DNA were strong at the periphery of the nucleolus at around 2 to 4 h PI (Fig. 3), suggesting that medusavirus DNA replication is initiated in the host cell nucleus. The amount of viral DNA increased significantly at 8 h PI and spread throughout the nucleus (Fig. 3). The cytoplasm of the host cells was filled with numerous capsids without DNA at 8 to 10 h PI, but the host cells maintained the integrity of the nuclear membrane even after viral infection (Fig. 4). At 14 h PI, signals of putatively newly synthesized medusavirus DNA were observed in the cytoplasm of the host cells and increased until 48 h PI (yellow arrows in Fig. 3). New virions were released from the host cells at 14 h PI, and a number of replicated virions were detected in the culture media around 29 h PI.

### The genome of medusavirus.

**FIG 5:**
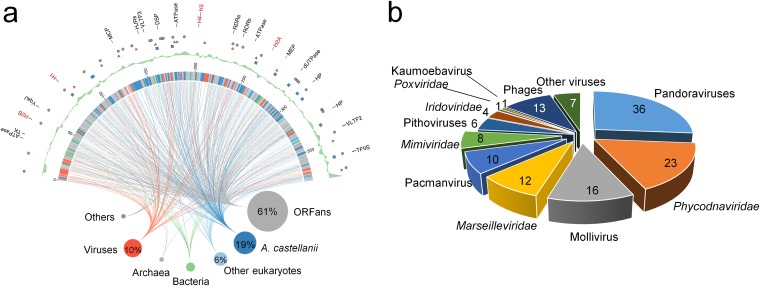
The rhizome and features of the medusavirus genome. (a) The rhizome (inner part of the figure) shows the organismal distribution of the closest homologs in the databases. The first outer ring shows the positions and names of NCLDV core genes (black) and histone genes (red). The second to third outer rings indicate the positions and inferred directions of laterally transferred genes (gray circle, direction undetermined; red triangle, medusavirus to A. castellanii; blue square, A. castellanii to medusavirus). The fifth outer ring shows G+C content skew over the 2.5-kb window. (b) BLASTP homology search of medusavirus ORFs against the viral database (E value < 1E−3). Numbers indicate the number of sequences of each NCLDVs possessing high homology with the medusavirus ORFs.

The genome of medusavirus was a linear 381,277-bp dsDNA. Its G+C content (61.7%) was the greatest among NCLDVs after pandoraviruses (62.0% on average) (Table 1) and slightly greater than that of the host amoeba genome (58.4%). We identified a total of 461 predicted protein-coding genes (open reading frames [ORFs]) and three tRNA-like sequences, corresponding to a theoretical coding density of 89.5% (Table S1). Among the predicted protein-coding genes, seven harbored putative spliceosomal introns (Table S1). Of all the ORFs, 182 (39%) showed homologs in the public sequence databases, including 115 closest homologs in eukaryotes, 45 in viruses, 18 in prokaryotes, and four in unclassified organisms, whereas 279 (61%) were ORFans (Fig. 5a). Notably, 86 predicted proteins had their closest homologs in A. castellanii strain Neff (Fig. 5a), suggesting that massive gene exchanges occurred between the ancestors of medusavirus and Acanthamoeba. There were 137 predicted proteins with significant sequence similarities to viral proteins in the databases, of which 117 proteins showed the closest viral homologs in other large DNA viruses, including 36 proteins in pandoraviruses, 23 proteins in Phycodnaviridae, and 16 proteins in mollivirus (Fig. 5b).

**TABLE 1:** G+C contents of NCLDVs

Family/group	No. of species	G+C content (%)				
		Maximum	Minimum	Mean	Median	SD
Pandoraviruses	3	63.66	60.66	62.01	61.72	1.52
Medusavirus	1	61.68	61.68	61.68	61.68	
Mollivirus	1	60.11	60.11	60.11	60.11	
*Iridoviridae*	37	55.37	27.23	46.33	53.92	11.04
*Ascoviridae*	5	49.66	35.23	45.17	45.87	5.85
Kaumoebavirus	1	43.70	43.70	43.70	43.70	
*Marseilleviridae*	12	44.73	42.69	43.49	43.04	0.82
*Phycodnaviridae*	24	55.00	30.37	43.35	41.92	5.49
*Asfarviridae*	4	38.95	38.59	38.77	38.76	0.17
Pithoviruses	2	41.49	35.80	38.65	38.65	4.03
Faustoviruses	8	37.76	36.21	36.84	36.48	0.73
*Poxviridae*	61	66.69	17.78	33.86	32.33	12.71
Pacmanvirus	1	33.62	33.62	33.62	33.62	
*Mimiviridae*	14	31.99	23.34	26.96	26.64	2.47

**FIG 6:**
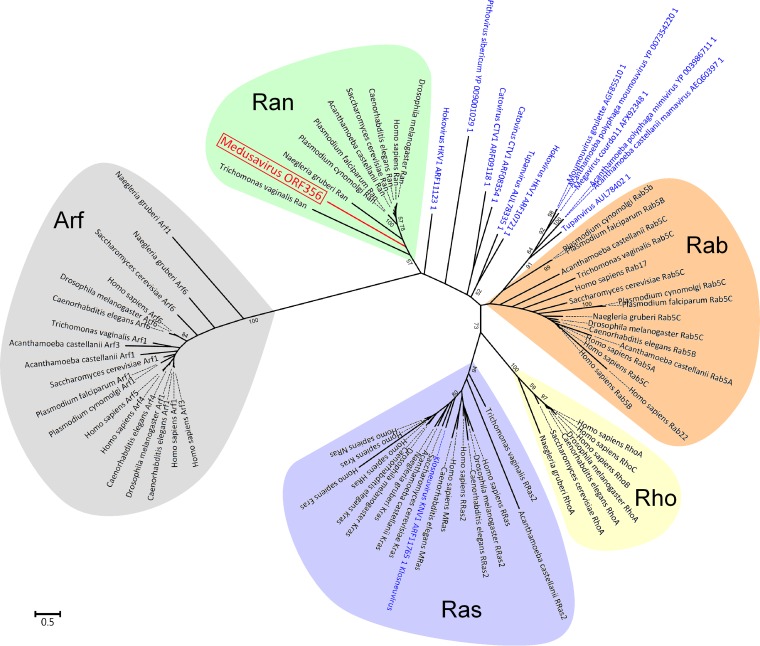
Phylogenetic tree of proteins in the Ras superfamily and their viral homologs. The red branch and label represent a medusavirus sequence. Blue labels represent sequences of other viruses. Subfamilies of eukaryotic proteins are shown by different colors, as follows: Arf (gray), Rab (brown), Ran (green), Ras (purple), and Rho (yellow). The scale bar indicates the expected number of amino acid substitutions per site.

We were able to assign putative functions to 105 (23%) ORFs. Predicted proteins encoded in the genome of medusavirus included a variety of enzymes for DNA/nucleotide metabolisms, such as a family B DNA polymerase (PolB), a DNA primase, three sliding clump proteins, an RNase HII, a Holliday junction resolvase, ribonucleotide reductase large and small subunits, a nucleoside diphosphate kinase, a thymidylate synthase, a deoxycytidylate deaminase, and a dUTPase (Table S1). In addition, medusavirus was predicted to encode several transcription-related proteins, such as a transcription elongation factor S-II, viral late transcription factors 2 and 3, a poly(A) polymerase regulatory subunit, and four homologs of Rho transcription termination factor (Table S1). Furthermore, we identified genes for the translation initiation factor eIF1 and a tRNA^His^ guanylyltransferase. Genes for the MCP and three variola virus (VV) A32-like DNA-packaging ATPases were readily identified. However, medusavirus had no genes for a DNA-dependent RNA polymerase, mRNA capping enzyme, or DNA topoisomerase II. All known NCLDVs encode one or more of these enzymes in their genomes, but even fragments or remnants of these genes were not detected in the medusavirus genome. The lack of these enzyme genes in medusavirus may be consistent with its putative dependence on the host nucleus for DNA replication. The genome of medusavirus was found to encode a homolog of GTP-binding Ras-related nuclear protein (Ran), which plays an important role in the directionality of nuclear import and export (55) (Fig. 6).

**FIG 7:**
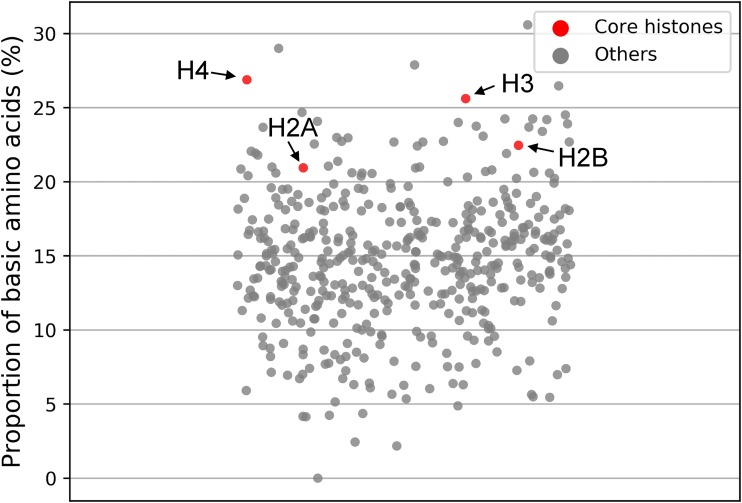
Proportion of basic amino acid residues in medusavirus predicted proteins. Core histone proteins show relatively higher proportion of basic amino acids.

**FIG 8:**
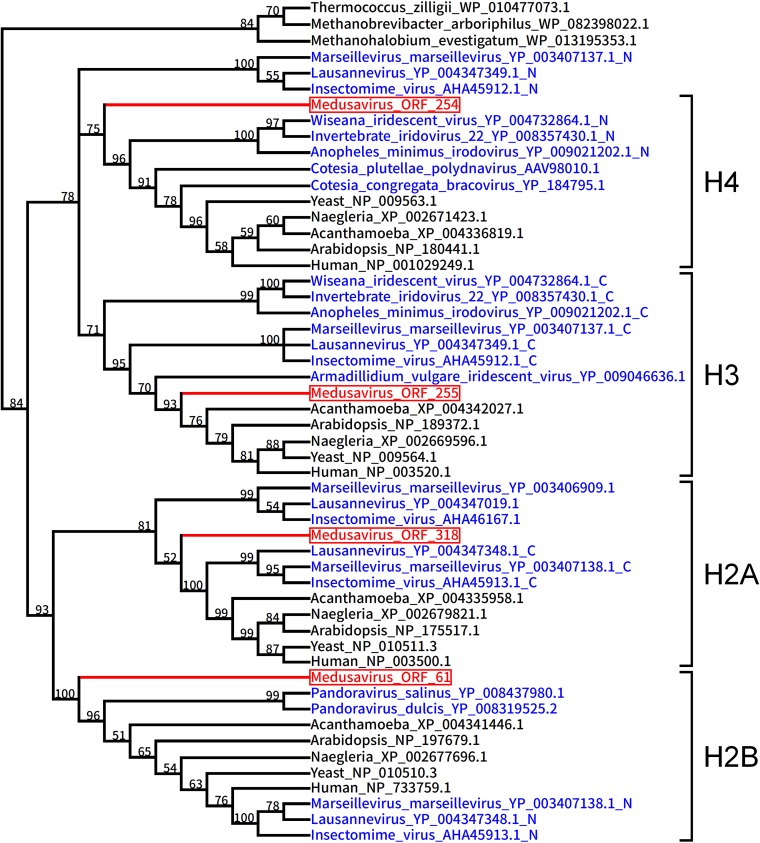
Bayesian phylogenetic tree of histone sequences. Red branches and labels represent medusavirus sequences. Blue labels represent genes of other viruses. Black labels represent archaeal or eukaryotic sequences. The branch length is not scaled. The human H2B sequence and some viral H2B sequences, such as Marseillevirus H2B, are grouped in this tree. However, the grouping is not supported by a high bootstrap value (76%) and is likely an artifact in tree reconstruction.

Medusavirus encoded homologs of all four core histones (i.e., H2A, H2B, H3, and H4) and the linker histone H1 (Fig. 5 and Table 2). In general, the core histone proteins are enriched with basic amino acid residues to facilitate interaction with the negatively charged DNA. Medusavirus core histone homologs were also enriched with basic amino acid residues (Fig. 7). Phylogenetic analysis of these core histone homologs revealed that the branches for medusavirus and other viral homologs were placed at the root of the respective core histone clades (Fig. 8).

**TABLE 2:** Histones encoded by viruses

Species/virus	No. of histones					Archaeal type	Total no. of genes^*a*^	Total no. of domain types
	H1	H2A	H2B	H3	H4			
Medusavirus	1	1	1	1	1		5	5
Brazilian marseillevirus		2	1	1		1	3	4
Port-miou virus		2	1	1		1	3	4
Lausannevirus		2	1	1		1	3	4
Kurlavirus BKC-1		2	1	1		1	3	4
Noumeavirus		2	1	1		1	3	4
Insectomime virus		2	1	1		1	3	4
Tunisvirus fontaine 2		2	1	1		1	3	4
Marseillevirus marseillevirus		2	1	1		1	3	4
Cannes 8 virus		2	1	1		1	3	4
Melbournevirus		2	1	1		1	3	4
Tokyovirus A1		2	1	1		1	3	4
Pandoravirus dulcis			1				1	1
Pandoravirus salinus			1				1	1
Armadillidium vulgare iridescent virus				1	1		1	2
Anopheles minimus irodovirus				1	1		1	2
Invertebrate iridovirus 22				1	1		1	2
Invertebrate iridescent virus 30				1	1		1	2
Invertebrate iridescent virus 22				1	1		1	2
Invertebrate iridovirus 25				1	1		1	2
Wiseana iridescent virus				1	1		1	2
Cotesia congregata bracovirus					1		1	1
Cotesia plutellae polydnavirus					1		1	1

a In Marseilleviridae and Iridoviridae, some histone domains are fused in a single gene.

**FIG 9:**
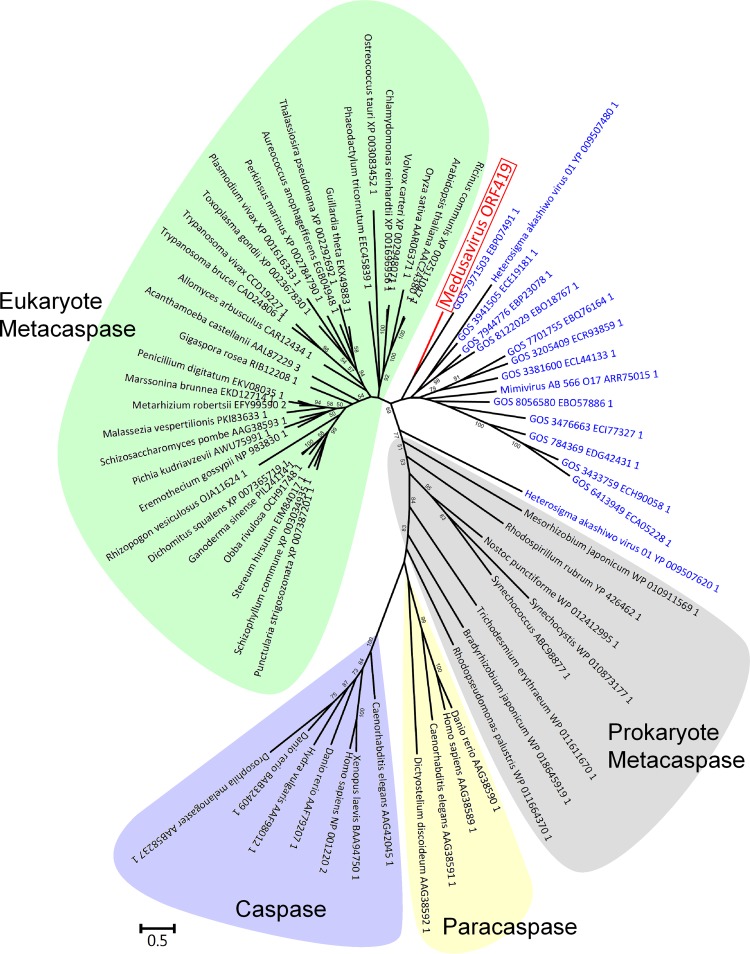
Phylogenetic tree of metacaspases. Red branch and label represent a medusavirus sequence. Blue labels represent sequences of other viruses. Eukaryotic and prokaryotic metacaspase proteins are shown by green and gray, respectively. Caspases and paracaspases are included as outgroups (purple and yellow, respectively). The scale bar indicates the expected number of amino acid substitutions per site.

The genome of medusavirus harbored other predicted proteins that were atypical for viral proteins. These include a cyclin B homolog, which may regulate the G2-M phase transition of the host amoeba (56, 57); a putative metacaspase; and a homolog of the mitochondrial chaperone BCS1. Putative viral metacaspases have previously been identified in an environmental giant virus single-amplified genome (gvSAG-566-O17) and marine metagenomes (58). As virus infection induces the host’s programmed cell death and the activation of host metacaspase in Emiliania huxyleyi (58–60), the virally encoded medusavirus metacaspase may serve to enhance the efficiency of infection by regulating programmed cell death and/or host stress responses. Our phylogenetic analysis of metacaspases revealed that the medusavirus metacaspase gene forms a monophyletic clade with the environmental sequences from ocean samples, including that of gvSAG-566-O17 (Fig. 9). We also found that a hypothetical protein (GenBank accession no. YP_009507480.1) of Heterosigma akashiwo virus 01 (HaV01) belongs to the same clade. To our knowledge, these medusavirus and HaV01 cases represent the first identification of putative metacaspase homologs encoded in cultured viruses.

### Proteome analysis of medusavirus virions.

Proteomic analysis of medusavirus virions revealed 80 virion proteins (Table 3). Among them, 54 (68%), including 20 ORFans, had unknown functions. Identified proteins included the MCP, two DNA-packaging ATPases, four Rho transcription termination factor homologs, multifunctional redox-active proteins such as glutaredoxin and thioredoxin homologs, and all virally encoded core histones, *viz*., H2A, H2B, H3, and H4. Although our proteomic analysis was not truly quantitative, the semiquantitative exponentially modified protein abundance index (emPAI) (61) values were relatively high for the core histone proteins (Table 3). Compared with the emPAI value of the MCP showing the highest emPAI value, the emPAI values were 28%, 21%, 9%, and 2.7% for H3, H2B, H4, and H2A, respectively. This suggests that these histones may be sufficiently abundant to package the viral DNA within the capsid.

**TABLE 3:** The proteome of medusavirus

ORF no.	Putative function	Molecular wt (kDa)	emPAI
177	Major capsid protein	54	24.470
63	Hypothetical protein	20	21.526
120	Hypothetical protein	42	9.380
432	Hypothetical protein	114	8.871
433	LYR motif-containing protein	114	8.208
255	Histone H3	18	6.906
149	High-mobility-group protein	11	5.268
61	Histone H2B	20	5.232
145	Ser/Thr protein kinase	82	4.125
249	Hypothetical protein	19	3.953
405	Hypothetical protein	19	3.953
72	Hypothetical protein	22	3.809
9	Hypothetical protein	27	3.467
361	Rho termination factor N-terminal domain-containing protein	56	3.354
28	Hypothetical protein	24	3.102
116	Rho termination factor N-terminal domain-containing protein	27	3.048
236	Hypothetical protein	70	2.722
8	Hypothetical protein	19	2.622
5	Rho termination factor N-terminal domain-containing protein	46	2.475
321	Rho termination factor N-terminal domain-containing protein	59	2.307
351	Hypothetical protein	24	2.292
445	Hypothetical protein	76	2.246
232	Hypothetical protein	38	2.240
254	Histone H4	10	2.203
182	Glutaredoxin	19	2.143
179	Hypothetical protein	262	2.107
413	Hypothetical protein	91	1.970
189	Hypothetical protein	14	1.963
74	Hypothetical protein	60	1.913
66	Hypothetical protein	23	1.898
442	Hypothetical protein	23	1.898
226	Putative membrane protein	40	1.807
239	Hypothetical protein	15	1.788
337	FG-GAP repeat-containing protein	74	1.578
339	Hypothetical protein	24	1.505
268	Hypothetical protein	50	1.456
454	PAN domain-containing protein	17	1.430
113	Hypothetical protein	29	1.394
1	Hypothetical protein	14	1.385
238	Hypothetical protein	14	1.369
341	PAN domain-containing protein	14	1.340
333	Hypothetical protein	24	1.182
421	Hypothetical protein	73	1.014
443	Hypothetical protein	24	0.894
262	Hypothetical protein	122	0.877
244	Putative tRNA-His guanylyltransferase	30	0.875
62	Hypothetical protein	25	0.873
180	Hypothetical protein	26	0.830
190	Hypothetical protein	96	0.829
281	Hypothetical protein	37	0.828
331	Hypothetical protein	77	0.787
111	Hypothetical protein	27	0.786
197	Hypothetical protein	45	0.758
458	Hypothetical protein	28	0.744
386	Hypothetical protein	17	0.720
338	Hypothetical protein	23	0.719
447	Hypothetical protein	23	0.719
200	Hypothetical protein	62	0.679
318	Histone H2A	25	0.663
349	Thioredoxin	19	0.620
122	Hypothetical protein	27	0.600
134	Hypothetical protein	14	0.565
241	Hypothetical protein	15	0.516
154	Hypothetical protein	15	0.507
307	Putative proliferating cell nuclear antigen	31	0.503
306	Hypothetical protein	54	0.422
329	Putative myristoylated membrane protein	27	0.418
229	Putative VV A32-like packaging ATPase	37	0.405
123	PKD^*a*^ domain-containing protein	28	0.398
103	Hypothetical protein	29	0.389
93	DUF4804 domain-containing protein	49	0.383
279	Ribonucleotide reductase large subunit	72	0.365
436	Hypothetical protein	23	0.305
332	Hypothetical protein	24	0.299
308	Hypothetical protein	78	0.281
256	Hypothetical protein	41	0.263
434	Hypothetical protein	43	0.248
340	Nucleoside diphosphate kinase	38	0.182
188	Hypothetical protein	105	0.096
310	Hypothetical protein	45	0.073

a PKD, polycystic kidney disease.

### Medusavirus represents a new lineage of large DNA viruses.

**FIG 10:**
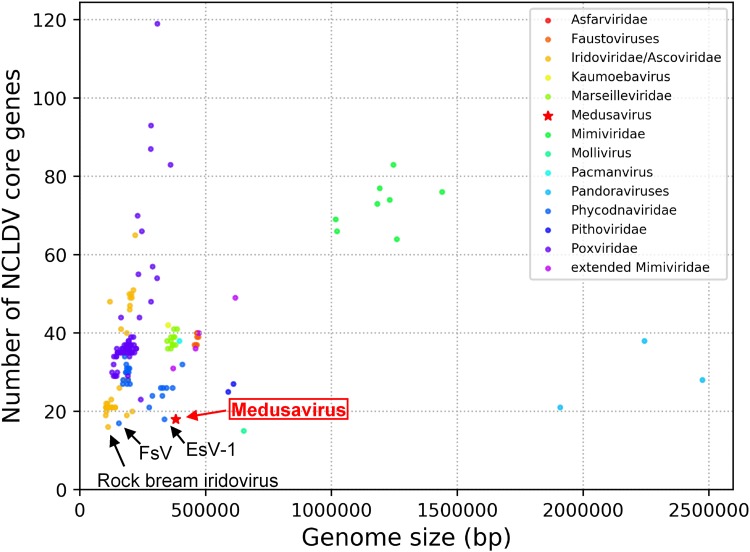
Number of NCLDV core genes. Long virus names have been replaced by acronyms. FsV, Feldmannia species virus; EsV-1, Ectocarpus siliculosus virus 1.

**FIG 11:**
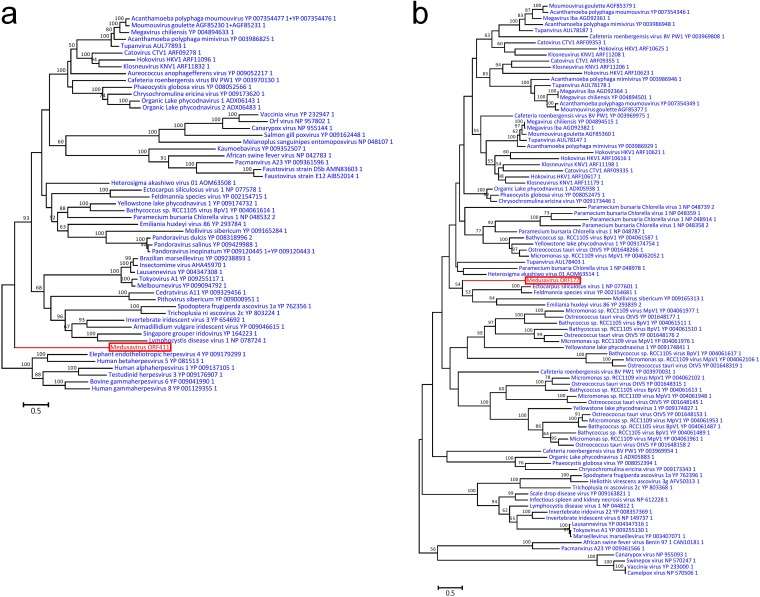
Maximum likelihood phylogenetic trees of viral proteins. Red branches and labels represent medusavirus sequences. Blue labels represent sequences of other viruses. The scale bars indicate the expected number of amino acid substitutions per site. (a) DNA polymerases. (b) Major capsid proteins.

**FIG 12:**
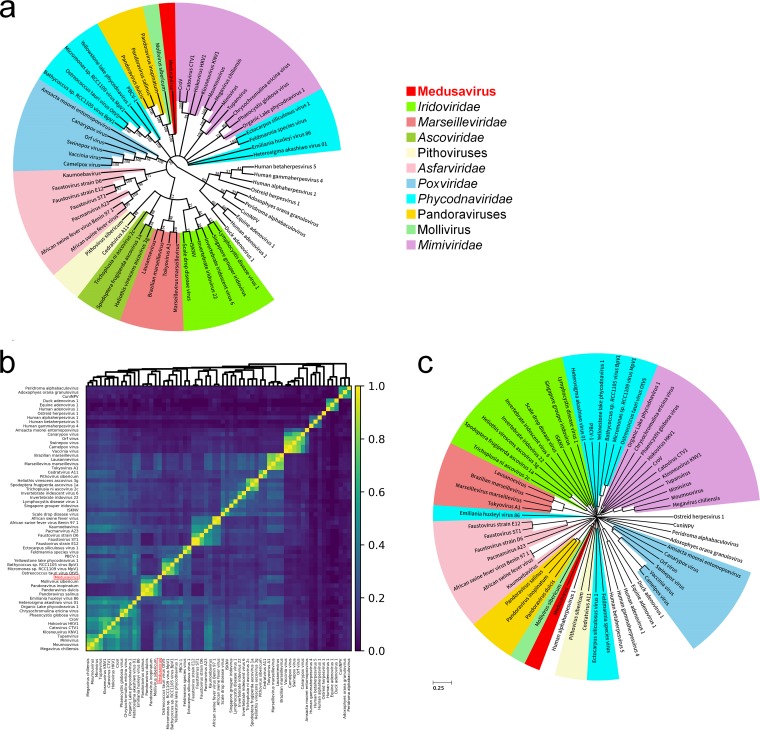
Phylogenomic relationships with other large DNA viruses. (a) Gene-content based cladistic tree. Each color represents a family or group of giant viruses. Long virus names have been replaced by acronyms. CroV, Cafeteria roenbergensis virus; PBCV-1, Paramecium bursaria Chlorella virus 1; ISKNV, Infectious spleen and kidney necrosis virus; CuniNPV, Culex nigripalpus nucleopolyhedrovirus. (b) Pairwise genomic similarity based on gene content. (c) Proteomic tree. Representations are the same as those in panel a.

The genome of medusavirus encoded 18 genes that were classified into 15 of the previously defined 47 NCLDV core genes (25) (Table 4). The number of NCLDV core genes in medusavirus is thus comparable to those found in other NCLDVs with a relatively small genome (e.g., Feldmannia species virus [155 kb, 17 core genes]; Rock bream iridovirus [112 kb, 16 core genes]) (Fig. 10). Phylogenetic analyses of DNA polymerases and MCPs did not support the inclusion of medusavirus in any of the existing groups or families of DNA viruses (Fig. 11). The gene content-based cladistics tree and proteomic tree also indicated that medusavirus represents an independent lineage among known DNA viruses, by branching from the root of the clade comprised of mollivirus and pandoraviruses (Fig. 12).

**TABLE 4:** NCLDV core genes found in medusavirus

NCVOG name	ORF no.	NCVOG annotation(s)^*a*^	Functional category(ies)
NCVOG0038	411	DNA polymerase elongation subunit family B	DNA replication, recombination, and repair
NCVOG0023	368, 409	D5-like helicase-primase	DNA replication, recombination, and repair
NCVOG0278	192	RuvC, Holliday junction resolvases (HJRs); poxvirus A22 family	DNA replication, recombination, and repair
NCVOG1192	71	YqaJ viral recombinase	DNA replication, recombination, and repair
NCVOG1164	420	A1L transcription factor/late transcription factor VLTF2	Transcription and RNA processing
NCVOG0262	196	Poxvirus late transcription factor VLTF3 like	Transcription and RNA processing
NCVOG0272	439	Transcription factor S-II (TFIIS)	Transcription and RNA processing
NCVOG0276	287	Ribonucleoside diphosphate reductase, beta subunit	Nucleotide metabolism
NCVOG1353	279	Ribonucleoside diphosphate reductase, alpha subunit	Nucleotide metabolism
NCVOG0320	41	Thymidylate kinase	Nucleotide metabolism
NCVOG1068	348	dUTPase	Nucleotide metabolism
NCVOG0022	177	NCLDV major capsid protein	Virion structure and morphogenesis
NCVOG0249	37, 229, 302	Packaging ATPase	Virion structure and morphogenesis
NCVOG0211	329	Myristoylated IMV envelope protein	Virion structure and morphogenesis
NCVOG0040	209	Dual-specificity phosphatases (DSP); Ser/Thr and Tyr protein phosphatases	Other metabolic functions

a VLTF, viral late gene transcription factor; IMV, intracellular mature virus.

### Medusavirus DNA polymerase is similar to the eukaryotic Pol δ.

**FIG 13:**
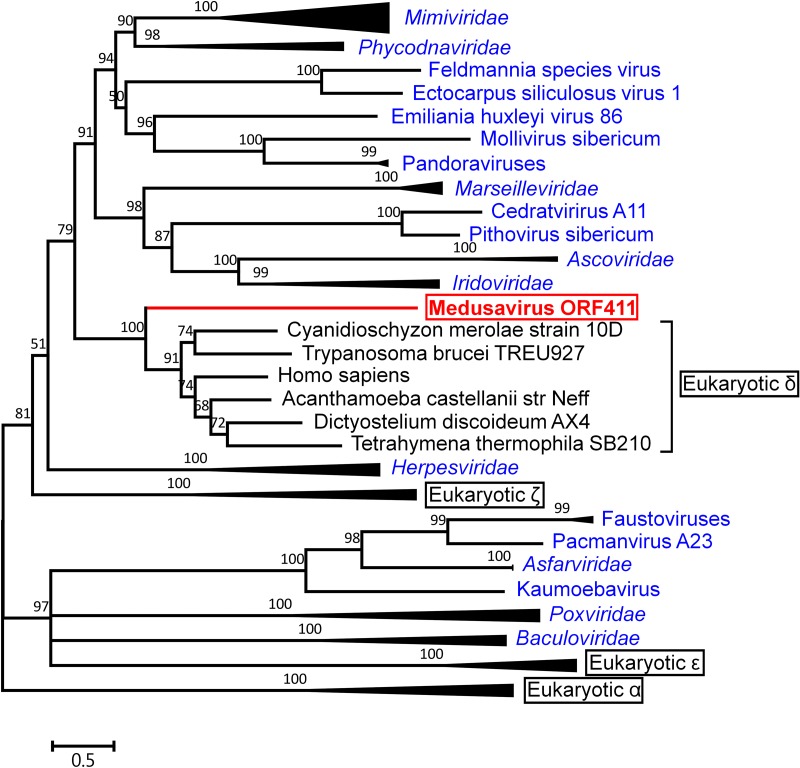
Bayesian phylogenetic tree of DNA polymerase sequences. The red branch and label represent a medusavirus sequence. Blue labels represent sequences of giant viruses. Black labels represent eukaryotic sequences. Eukaryotic DNA polymerases α, δ, ε, and ζ were abbreviated to Eukaryotic α, Eukaryotic δ, Eukaryotic ε, and Eukaryotic ζ, respectively. The scale bar indicates the expected number of amino acid substitutions per site.

The reconstructed tree placed medusavirus PolB at the root of, but not inside, the eukaryotic DNA polymerase delta (Pol δ) clade (Fig. 13). Other viral PolB sequences were clearly separated from medusavirus PolB in the reconstructed phylogenetic tree (Fig. 13).

### Lateral gene transfers between medusavirus and amoeba.

**FIG 14:**
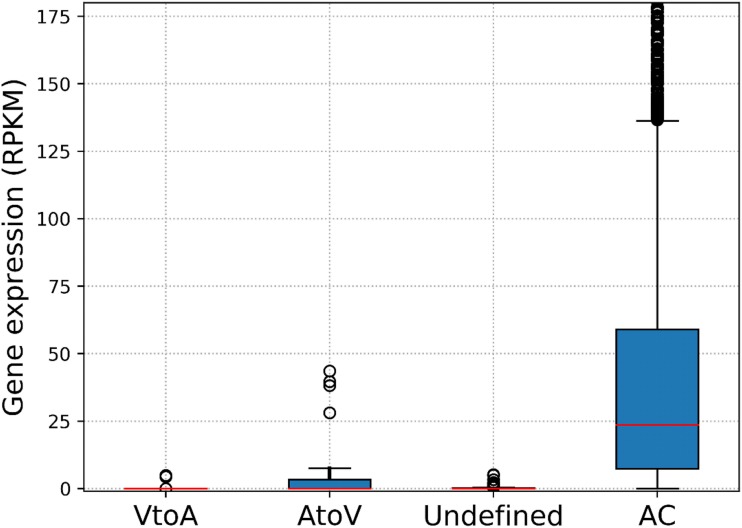
Transcriptional activity expressed as RPKM (number of reads per kilobase per million reads) of the LGT candidate genes in A. castellanii. “VtoA,” “AtoV,” and “undefined” represent LGT genes and their predicted directions. “AC” represents genes conserved among the species of Amoebozoa.

By reciprocal BLAST searches, we identified 57 LGT candidate genes between medusavirus and A. castellanii. 13 of the 57 genes were predicted to be transferred from virus to amoeba (VtoA) and 12 were in the reverse direction (AtoV) (Table 5). The directions for the remaining 32 cases could not be determined (Table 5). AtoV genes included a linker histone H1 gene, while VtoA genes included several viral hallmark genes, such as an MCP gene and a DNA-packaging ATPase gene. LGT candidates with undetermined directions were enriched with hypothetical proteins. We analyzed the transcriptional activities expressed as RPKM (number of reads per kilobase per million reads) of the LGT candidate genes in A. castellanii. The LGT candidate genes showed lower transcriptional activities than other genes conserved among the species of Amoebozoa (i.e., “vertically inherited genes”) (Mann-Whitney U test, *P* = 2.00 × 10^−60^) (Fig. 14). Transcriptional activities of both VtoA genes and LGT candidate genes with undetermined directions were even lower than those of AtoV genes (*P* = 0.026), suggesting that some of these genes are no longer functional or are silenced in the amoeba genome. This is consistent with the previous observation that approximately half of A. castellanii genes putatively acquired from large DNA viruses are transcriptionally inactive (62).

**TABLE 5:** LGT genes between medusavirus and A. castellanii

Medusavirus ORF no.	Putative function	A. castellanii gene accession no.	Prediction of direction^*a*^
330	DUF4326 domain-containing protein	XP_004336203.1	AtoV
349	Thioredoxin	XP_004340322.1	AtoV
375	DNA helicase	XP_004347213.1	AtoV
309	F-box domain-containing protein	XP_004337331.1	AtoV
412	Ser/Thr protein kinase	XP_004347234.1	AtoV
106	Linker histone H1	XP_004337841.1	AtoV
224	Ribonuclease HII	XP_004332877.1	AtoV
138	PIN domain-containing protein	XP_004339581.1	AtoV
139	BTB/POZ^*b*^ domain-containing protein	XP_004356555.1	AtoV
439	Transcription elongation factor S-II	XP_004339821.1	AtoV
204	BTB/POZ domain-containing protein	XP_004334604.1	AtoV
365	Replication factor C large subunit	XP_004357147.1	AtoV
111	Hypothetical protein	XP_004334601.1	VtoA
329	Putative myristoylated membrane protein	XP_004339071.1	VtoA
429	Hypothetical protein	XP_004335825.1	VtoA
188	Hypothetical protein	XP_004336714.1	VtoA
308	Hypothetical protein	XP_004335814.1	VtoA
261	Hypothetical protein	XP_004339028.1	VtoA
226	Putative membrane protein	XP_004339025.1	VtoA
454	PAN domain-containing protein	XP_004339817.1	VtoA
229	Putative VV A32-like packaging ATPase	XP_004335550.1	VtoA
177	Major capsid protein	XP_004336719.1	VtoA
203	Molybdenum cofactor carrier	XP_004340814.1	VtoA
282	Class 3 lipase	XP_004339082.1	VtoA
154	Hypothetical protein	XP_004347217.1	VtoA
210	Hypothetical protein	XP_004333492.1	Undetermined
351	Hypothetical protein	XP_004347207.1	Undetermined
354	Hypothetical protein	XP_004347218.1	Undetermined
199	Hypothetical protein	XP_004338290.1	Undetermined
200	Hypothetical protein	XP_004335813.1	Undetermined
290	Hypothetical protein	XP_004339813.1	Undetermined
186	Hypothetical protein	XP_004336716.1	Undetermined
236	Hypothetical protein	XP_004335548.1	Undetermined
134	Hypothetical protein	XP_004335826.1	Undetermined
310	Hypothetical protein	XP_004339029.1	Undetermined
178	Hypothetical protein	XP_004336720.1	Undetermined
179	Hypothetical protein	XP_004335539.1	Undetermined
74	Hypothetical protein	XP_004337837.1	Undetermined
103	Hypothetical protein	XP_004339001.1	Undetermined
5	Rho termination factor N-terminal domain-containing protein	XP_004337835.1	Undetermined
407	Hypothetical protein	XP_004340313.1	Undetermined
403	Hypothetical protein	XP_004335821.1	Undetermined
404	Hypothetical protein	XP_004352648.1	Undetermined
405	Hypothetical protein	XP_004347220.1	Undetermined
23	Hypothetical protein	XP_004338755.1	Undetermined
225	Hypothetical protein	XP_004339084.1	Undetermined
156	F-box domain-containing protein	XP_004335809.1	Undetermined
213	Hypothetical protein	XP_004333493.1	Undetermined
214	Hypothetical protein	XP_004333494.1	Undetermined
438	Hypothetical protein	XP_004339822.1	Undetermined
285	Hypothetical protein	XP_004340318.1	Undetermined
369	Hypothetical protein	XP_004339083.1	Undetermined
357	Hypothetical protein	XP_004347215.1	Undetermined
452	Hypothetical protein	XP_004352718.1	Undetermined
421	Hypothetical protein	XP_004335432.1	Undetermined
281	Hypothetical protein	XP_004334602.1	Undetermined
62	Hypothetical protein	XP_004337997.1	Undetermined

a AtoV, amoeba to virus; VtoA, virus to amoeba.

b BTB, BR-C, ttk and bab; POZ, Pox virus and zinc finger.

**FIG 15:**
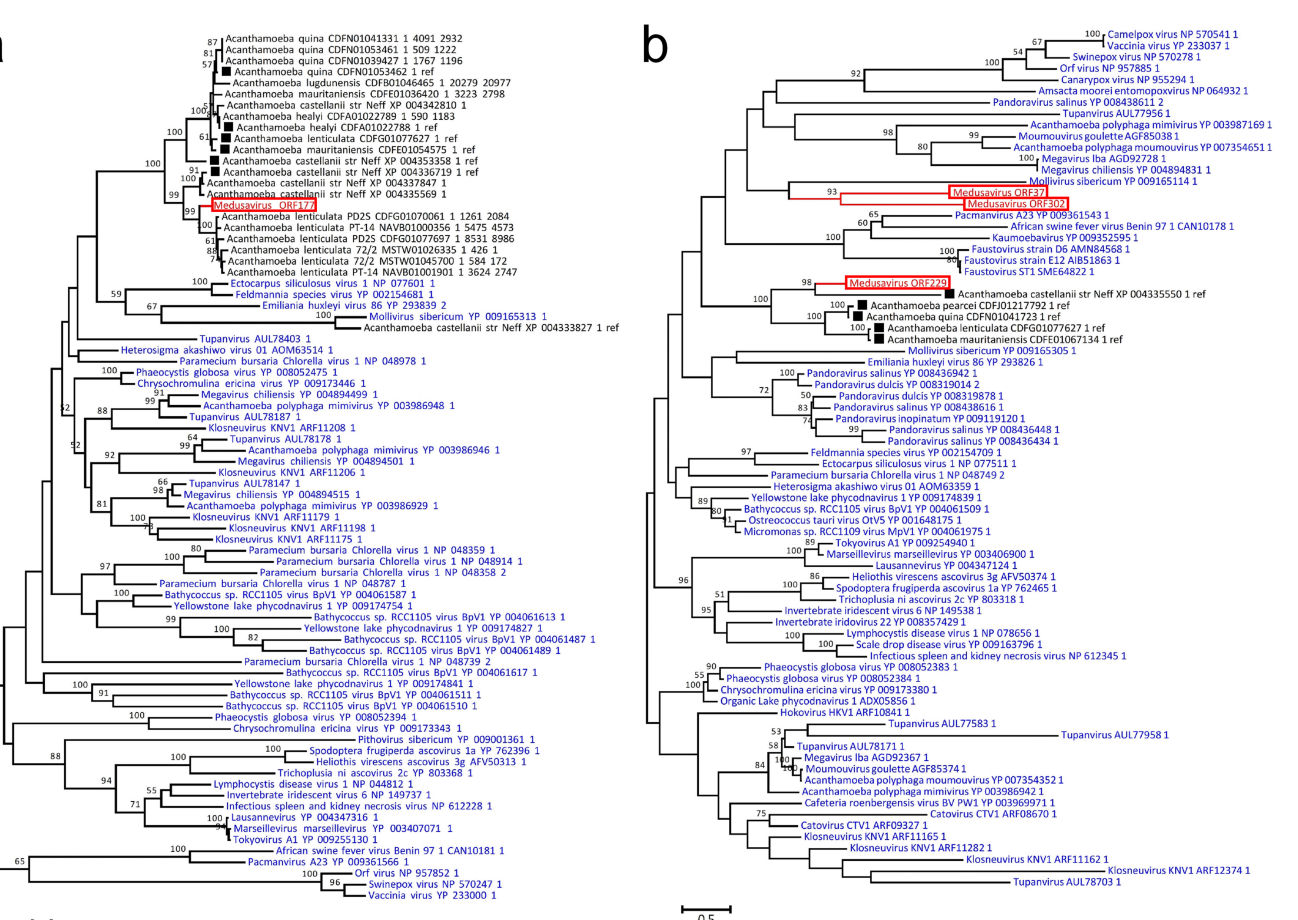
Maximum likelihood phylogenetic tree of homologs of LGT candidate genes. Red branch and label represent a medusavirus sequence. Blue labels represent sequences of giant viruses. Black labels represent sequences encoded in Acanthamoeba genomes. Squares represent proteins which were referenced from previous research. The scale bars indicate the expected number of amino acid substitutions per site. (a) Major capsid proteins. (b) DNA-packaging ATPases.

Recent studies have revealed the presence of NCLDV MCP genes in the genomes of Acanthamoeba spp. (63, 64), suggesting gene transfers from viruses to Acanthamoeba. Phylogenetic analyses indicated that some of these sequences formed unidentified NCLDV clades (62, 64, 65). Therefore, we performed phylogenetic reconstructions of MCPs and DNA-packaging ATPases, including homologs from the genomes of medusavirus and Acanthamoeba spp. The results indicated that medusavirus protein sequences form monophyletic groups with the previously identified homologs in Acanthamoeba (Fig. 15).

## DISCUSSION

Previous metagenomic analyses indicated that giant viruses could inhabit heated environments, such as hot deserts and hot springs (66, 67), although no giant virus had been isolated from such special environments. Medusavirus is the first giant virus isolated from a heated environment (43.4°C), and it shows several unique features in its replication cycle and particle morphology. It also presented distant phylogenetic and genomic relationships with other known large DNA viruses. Therefore, we propose that medusavirus represents a new family of large DNA viruses, Medusaviridae.

Single-particle cryo-EM revealed that the medusavirus shows structural features common to other icosahedral NCDLVs. The internal membrane surrounding the viral DNA is a typical feature of all structurally characterized icosahedral NCDLVs (68). The internal membrane of medusavirus extends and directly binds to the major capsid below the 5-fold axis (arrows in Fig. 2b), whereas it swells near the 5-fold axis in Melbournevirus (MelV) of the family Marseilleviridae (69). The total particle size of 260 nm of medusavirus, including the 14-nm surface spikes, is larger than that of MelV. However, the actual capsid diameter, 232 nm (excluding the 14-nm surface spikes) is similar to that of MelV, although T=277 of the medusavirus capsid is smaller than T=304 of the MelV capsid. The average distance between the MCPs was estimated to be 7.55 nm for medusavirus and 7.44 nm for MelV. Therefore, the MCPs are somewhat more loosely packed in medusavirus than in MelV. Faustovirus has been previously reported as a T=277 icosahedral large DNA virus (70). The virus has a larger capsid diameter (240 nm) than the actual capsid diameter (232 nm) of medusavirus excluding the 14 nm surface spikes. The faustovirus virion has a double layered capsid, where the packing of the outer shell can be influenced by the inner shell formed with a T=64 icosahedron.

The most unique structural feature of medusavirus is the presence of spherical-headed spikes on the capsid surface. Spike structures on the capsid surface have been reported for several NCLDVs, such as Paramecium bursaria Chlorella virus (PBCV-1) and Phaeocystis pouchetii virus (PpV01), but their locations on the capsid surface are limited (71). Our cryo-EM results suggest that the T=277 icosahedral capsid of medusavirus is covered with 2,660 spikes, assuming that each capsomer has one spike. Chilo iridescent virus (CIV) also has short fibers that extend from each capsomer. The number of CIV fibers is estimated at 1,460, based on the T=185 icosahedral capsid (71). However, CIV fibers appear to be more flexible and do not exhibit a spherical-headed structure, unlike the medusavirus spikes.

A notable feature of the replication cycle of medusavirus is the entry of the viral genome into the host nucleus, eventually filling the nucleus with the synthesized viral DNA. Our FISH analysis showed that viral DNA replication was initiated inside the nucleus at the periphery of the nucleolus and appeared to be completed in the nucleus (Fig. 3). Several NCLDVs transfer the viral DNA to the host nucleus to initiate DNA replication. Iridoviridae and Asfarviridae replication cycles are initiated in the nucleus but are completed in the cytoplasm (72). In the case of a Phycodnaviridae PBCV-1, the viral DNA, and probably DNA-associated proteins, move to the nucleus, where early transcription is detected within 5 to 10 min PI (73). The replication cycles of pandoraviruses and mollivirus involve the disorganization or deformation of the nucleus, respectively, suggesting that their early replication phase depends on host nuclear functions (14, 32). Marseilleviruses replicate in the cytoplasm, which initiate their replication by transiently recruiting the nuclear transcription machinery to their cytoplasmic viral factory (74). Thus, there appears to be a variety of dependences on the host nuclear functions across giant viruses. Medusavirus was found to encode neither an RNA polymerase nor DNA topoisomerase II, although all known NCLDVs encode at least one of these enzymes. DNA topoisomerase II encoded by PBCV-1 is thought to function in the late stages of viral replication or packaging, both of which occur in the cytoplasm (75, 76). Medusavirus may be recruiting these functions from the host. The presence of spliceosomal intron-like sequences and the lack of an mRNA capping enzyme gene suggest that medusavirus may also be dependent on the host nucleus for mRNA processing.

In addition, medusavirus provided us the answer to the enigmatic presence of the MCP genes in the Acanthamoeba genome (Fig. 15a). Previous studies predicted the existence of unidentified families of NCLDVs through the discovery of MCP genes in Acanthamoeba genomes (62–64). Our phylogenetic analysis shows that the medusavirus MCP gene forms a monophyletic group with the MCP genes in the amoeba genome and thus indicates that medusavirus indeed belongs to the predicted family. These observations clearly show that LGT of the MCP genes had occurred from medusavirus to Acanthamoeba in ancient times.

Furthermore, we detected traces of massive LGTs between medusavirus and Acanthamoeba in both the host-to-virus and virus-to-host directions. The entrance of the medusavirus genome into the nucleus may facilitate physical contact between the viral DNA and host DNA, possibly increasing the chance of LGT between medusavirus and Acanthamoeba. A number of viruses have already been isolated in laboratories using the amoeba coculture method, but from the natural environment no virus has been isolated with convincing evidence that allows a claim that Acanthamoeba is its genuine natural host. Medusavirus encodes a larger number of Acanthamoeba gene homologs (86/461 = 18.7%) than Mollivirus sibericum (50/523 = 9.6%) or Pandoravirus salinus (56/2336 = 2.4%) does (32). The significant amount of gene transfers observed between medusavirus and Acanthamoeba suggests that Acanthamoeba or a related amoeba is indeed the major natural host of medusaviruses.

Medusavirus was found to be the first isolated virus to encode all four core histone proteins and one linker histone domain. The four core histones were identified in virion proteomic analysis, suggesting their involvement in the viral DNA packaging and their possible formation of nucleosome-like structures in the medusavirus virion. The presence of the core histone genes has previously been reported in several other eukaryotic dsDNA viruses. In bracoviruses, the H4 protein plays a critical role in suppressing host (insect) immune responses during parasitism (77). Marseilleviruses are known to encode three sets of fused histone genes, H2B/H2A, archaeal histone/H3, and an unknown domain/H2A. These histones have also been found in marseillevirus virions (29) and are suggested to function in the compaction, protection, and/or regulation of the viral genomes (78). If the DNA replication, transcription, and mRNA capping of medusavirus are partly dependent on the host cell nucleus, as suggested above, the histones may also facilitate these processes via interaction with the host molecular machinery.

Based on the phylogenetic analysis of DNA polymerases, Villarreal and DeFilippis proposed a hypothesis that the DNA polymerase gene of an ancient DNA virus related to the extant Phycodnaviridae (Feldmannia sp. virus) gave rise to the eukaryotic Pol δ (39). Subsequent studies have revealed that Pol δ is closely related to PolBs of Phycodnaviridae, Mimiviridae, and pandoraviruses (41, 79). In the present study, medusavirus PolB has established another branch that is most closely related to the eukaryotic Pol δ clade but is clearly separate from PolBs of other known NCLDVs. In this reconstructed tree, the eukaryotic Pol δ clade was embedded inside a larger tree of viral homologs defining several outgroups. This tree topology suggests that the eukaryotic Pol δ originated from an ancestor of medusavirus or its relative. The phylogenetic tree of the medusavirus core histone homologs shows a similar tree topology, implying that eukaryotic histones may have derived from the ancient viruses through virus-to-eukaryote LGT. It is worth noting that dinoflagellates, which have largely abandoned histones, have apparently acquired the viral-derived alternatives for histones (80). Nonetheless, the possibility of the reverse host-to-virus transfer direction is not excluded for these putative LGTs.

Medusavirus is a novel large DNA virus isolated from hot spring water in Japan. Structural, genomic, and proteomic characterization of medusavirus revealed its unique features compared to other known large DNA viruses. Phylogenetic analyses suggest that the medusavirus lineage emerged in ancient times, but the virus presently encodes a full set of histone genes and a DNA polymerase gene, which are associated with modern eukaryotic homologs. On the other hand, the host amoeba encodes medusavirus homologs, including MCP. Taking these observations in account, we conclude that amoebae are the most promising natural hosts of medusavirus and that LGTs have occurred repeatedly and bidirectionally between medusavirus and its host due to physical contact between viral and host DNAs since ancient times. Medusavirus is the first NCLDV to be isolated from a thermal environment. This indicates that the ecological niche of NCLDVs is broader than previously thought. We would like to continue analyzing Medusaviridae, such as more detailed infection mechanisms, thermal tolerance, and diversity, etc. Further investigation of large DNA viruses should reveal the active coevolutionary interactions between the NCLDVs and eukaryotic organisms at the global scale.

## MATERIALS AND METHODS

### Virus isolation.

Acanthamoeba castellanii (Douglas) strain Neff (ATCC 30010) cells were purchased from the American Type Culture Collection (ATCC, Manassas, VA) and cultured in peptone-yeast-glucose (PYG) medium at 26°C as described previously (14, 81). An outflow water/soil sample (50 ml) was collected from a water sample spilled out from a hot spring in Japan. The water temperature was 43.4°C at the sampling site. After removal of floating bacteria and small viruses by filtration using a 20-μm filter (no. 43; Whatman International, Maidstone, UK), the collected mud and dead leaves were resuspended in 13 ml of sterile phosphate-buffered saline (PBS) and stirred gently for 1 day at room temperature. The sample was again filtered through another 20-µm filter. Then, the filtered sample (10 ml) was further filtered through a sterile 1.2-µm filter (Millex-AA; Merck Millipore, Darmstadt, Germany). The filtrate (9.5 ml) was then mixed with PYG medium (18 ml). Acanthamoeba cell suspension (0.5 ml) was added and incubated with gentle stirring for 1 h at room temperature, followed by incubation at 26°C in a total of 142 wells using two 96-well microplates. After 5 days, amoeba cells with delayed proliferation were screened. Culture supernatant from growth-retarded wells showing phenotypical difference was inoculated into fresh amoeba cells in an individual well of a 12-well microplate. After 3 days, supernatant of all three wells with cell encystment was inoculated into a fresh amoeba cell suspension in three 25-cm^2^ culture flasks and then in three 75-cm^2^ culture flasks. Supernatant from each 75-cm^2^ culture flask was stored at 4°C as an isolated virus solution (named HS-1, HS-2, and HS-3).

### Virus cloning and cultivation.

Among the three isolated virus solutions, virus cloning of HS-1 was performed according to a cloning method used for Mollivirus sibericum (32) with several modifications as described below. Briefly, HS-1-infected amoeba cells in 75-cm^2^ culture flask were harvested and washed with an excess amount of fresh PYG medium to remove surplus viruses. Amoeba cells were then resuspended in 16 ml of fresh PYG medium. Eight serial 3-fold dilutions were performed in a 96-well microplate by mixing 50 μl of the solution from the previous well with 100 μl of fresh PYG. Each last eighth dilution was examined under a light microscope to verify the existence of fewer than two amoeba cells per well. Only one amoeba cell was observed in each well. Several hundred fresh amoeba cells were added to the wells containing only one cell and cultured for 3 days until most cells exhibited encystment. The obtained viral clone was designated “Acanthamoeba castellanii medusavirus (Medusavirus),” and amplified and stored at 4°C for further use.

To routinely culture medusavirus, amoeba cells were initially cultured using eight 25-cm^2^ culture flasks, each containing 25 ml of PYG medium. The cells were inoculated with medusavirus (multiplicity of infection [MOI], ∼1 to 2), and then the culture media containing medusaviruses were harvested 1–4 days postinfection (PI). Amoeba cells and cell debris were removed by centrifugation (800 × *g*, 5 min, 24°C), and the medusavirus particles were collected by centrifugation (8,000 × *g*, 35 min, 4°C). The collected medusavirus particles were resuspended in 5 ml of PBS, and filtered through a 0.45-μm filter (Millex-AA, Merck Millipore, Darmstadt, Germany), centrifuged (8,000 × *g*, 35 min, 4°C), and resuspended in 10 μl of PBS. This purification protocol was performed 5 to 10 times to obtain high numbers of medusavirus particles.

### Cryo-electron microscopy and single-particle analysis.

A suspension of 2.5 ml of purified medusavirus particles was applied to an R1.2/1.3 Mo grid (Quantifoil Micro Tools GmbH, Germany), which was previously glow-discharged, and snap-frozen in liquid ethane using a Vitrobot Mark IV unit (FEI Company, USA) at a condition of 95% humidity at 4°C. Frozen grids were imaged using a JEM-2200FS electron microscope operated at 200 kV accelerating voltage and equipped with an omega-type energy filter and field emission electron source (JEOL Ltd., Japan). The images were recorded on a DE20 direct detector (Direct Electron LP, USA) at a nominal magnification of **×**25,000 for a 3-s exposure time with 75 movie frames. The total electron dose was below 20 electrons (e^−^)/Å^2^ for each image. The numerical pixel size corresponds to 2.3 Å on the specimen. The movie frames were motion corrected using a manufacture-provided script, DE_process_frames.py, and summed. The resulting images were subjected to single**-**particle analysis.

For single-particle analysis, a total of 5,406 medusavirus particles were selected from 1,198 motion-corrected images and binned by two using RELION software (82). The extracted particles were classified by reference-free alignment, where the class averages were simultaneously separated into DNA-filled, partially DNA-filled, filled with non-DNA, and empty particles classes. For structural analysis of the viral capsid, a total of 2,288 DNA-filled particles and a total of 1,397 empty particles were selected from well-aligned two-dimensional (2D) classes, respectively, and used for three-dimensional (3D) reconstruction by imposing the icosahedral symmetry. The handedness of the 3D map was determined by independent subtomogram averaging. The final map resolutions were estimated using the gold-standard Fourier shell correlation (GS-FSC) criterion of 0.143. The cryo-EM maps were visualized and annotated by UCSF Chimera (83). The icosahedral T-number was determined by manually counting the surface spike-like short fibers that extended from each capsomer.

### Conventional electron microscopy.

Harvested cells infected by medusaviruses (8 h PI) or purified medusavirus particles were subjected to regular transmission electron microscopic observation as described previously (81). Plastic-embedded virus-infected amoeba cells were sectioned at 70-nm thickness using an ultramicrotome (EM-UC7; Leica Microsystems, Austria). The thin sections were mounted on a Formvar-coated slot mesh and stained with 2% uranyl acetate and 1% lead citrate for 5 min each. Transmission electron microscopy observation was done using a JEM1010 microscope (JEOL Ltd., Japan) at 80 kV accelerating voltage. The images were recorded in a 2k × 2k side-mount Veleta charge-coupled device (CCD) camera (Olympus, Japan).

### Fluorescent *in situ* hybridization (FISH) analysis.

For tracing medusavirus DNA in host cells after infection, FISH analysis was performed as described below. Briefly, purified medusavirus DNA (4.68 μg) was labeled with Cy3 using the nick translation method. Amoeba cells cultured in a 12-well plate were infected with medusaviruses and harvested at 10 min, 30 min, 1 h, 2 h, 4 h, 8 h, 14 h, 24 h, and 48 h PI from each individual well of the 12-well plate. Cells were washed twice with PBS and fixed with methanol:acetate (3:1) solution. One drop of the fixed cell suspension was placed on a glass slide and air dried completely. Cy3-labeled medusavirus DNA probe was placed on the glass slide and incubated at 67°C for 5 min, followed by hybridization at 37°C for 2 h, and stringent washing with 50% formamide in 2× and 1× SSC buffer (1× SSC is 0.15 M NaCl plus 0.015 M sodium citrate). Cells on the glass slide were also stained with 4′,6-diamidino-2-phenylindole (DAPI). The detection of FISH and DAPI signals were performed using the Leica CW-4000 cytogenetic workstation (Leica Microsystems K.K., Tokyo, Japan).

### Genome analysis.

After virus cloning and purification, the genomic DNA of medusavirus (1.2 μg) was prepared using NucleoSpin tissue XS (Macherey-Nagel, Germany), following the manufacturer’s protocol, and further purified using AMPure XP (Beckman Coulter). The DNA library for sequencing was prepared using a g-Tube (Covaris) and an SMRTBell template prep kit 1.0 (Pacific Biosciences), and sequencing was performed on a PacBio RS II sequencer (Pacific Biosciences). The total number of subreads was 304,607, and the total number of sequenced nucleotides was 1,325,027,506. Canu v1.5 (84) was used to assemble the reads to generate a final single contig of 381,277 bp.

### Gene prediction and annotation.

Gene prediction was performed using GeneMarkS v4.32 (85). Putative introns were predicted using GeneWise v2.4.1 (86) with visual inspection of the alignments. Amino acid sequence similarity searches (E value < 1E−5) were performed against the UniRef90 database, Virus-Host Database (Virus-Host DB) (87), RefSeq database, and the Conserved Domains Database (CDD) using BLASTP and RPS-BLAST of BLAST+ (88) (v2.6.0). tRNA genes were identified using tRNAscan-SE (89) (v1.3.1). NCLDV core genes were assigned to viral genomes through homology searches against the Nucleo-Cytoplasmic Virus Orthologous Groups (NCVOG) database (90).

### Phylogenetic analysis.

The hidden Markov model (HMM) profiles for DNA polymerases, MCPs, and DNA-packaging ATPases were constructed using sequences in NCVOG (90). Homologs of each protein were identified using HMMsearch (91) against the Virus-Host DB (87). Sequences were aligned using Multiple Alignment using Fast Fourier Transform (MAFFT) v7.220 (92) with default parameters. Tree reconstruction was performed using RaxML v8.2.4 (93) with the selected LG+F model and PROTGAMMA parameter with 100 bootstrap replicates. The HMM profiles of histone families were constructed using sequences in the Kyoto Encyclopedia of Genes and Genomes (KEGG) Orthology (KO) database (94) (H2A, KO no. K11251; H2B, no. K11252; H3, no. K11253; and H4, no. K11254). The homologs of each protein were recruited using HMMsearch (91) against the Virus-Host DB (87). Eukaryotic and archaeal histone and eukaryotic DNA polymerase sequences were manually collected. Tree reconstruction was performed using PhyloBayesMPI (95) with four chains for at least 4,000 cycles. The cladistic tree was computed using the neighbor-joining method based on the presence/absence matrix of gene clusters derived from OrthoFinder (96) clustering with a previously proposed similarity score (97). Branch support values were estimated using 100 times of bootstrap resampling. The proteomic tree was computed using ViPTreeGen (98) (v1.1.0).

### Lateral gene transfer (LGT) analysis.

To identify LGT candidates between A. castellanii and medusavirus, bidirectional BLASTP searches were performed by including sequences from UniRef90 but excluding the query genome. UniRef90 contains the proteome sequences of A. castellanii strain Neff but does not contain most of the protein sequences from draft genome sequences of other A. castellanii strains. When a gene of A. castellanii got a best hit for a gene of medusavirus and the same medusavirus gene got a best hit for the same A. castellanii gene, the pair of genes were considered a candidate for LGT. For inference of the directions of the LGT candidates, the most similar homologs of the bidirectional BLASTP searches were examined after excluding the hits against A. castellanii or medusavirus genes. In the BLASTP result with a query of a medusavirus gene, the best-hit gene after excluding hits to A. castellanii genes was determined and considered to be the closest gene. In the same way, in the BLASTP result with a query of an A. castellanii gene, the best-hit gene after excluding hits to medusavirus genes was considered to be another closest gene. In this way, we defined the two closest genes for a pair of LGT candidates. If at least one of the closest genes was a viral gene, it was inferred that LGT occurred from virus to amoeba (VtoA). Conversely, if at least one of the closest genes was a eukaryote gene, it was inferred that LGT occurred from amoeba to virus (AtoV). In other cases, we did not determine the direction of LGT. The transcriptional activity of the candidate LGT genes was determined using the transcriptome sequencing (RNA-seq) data sets of A. castellanii in the GenBank Sequence Read Archive (SRA), namely accession no. SRR611709, SRR611787, SRR611788, SRR611790, SRR611791, SRR611792, SRR611793, SRR611795, SRR611796, SRR611797, SRR629488, SRR957287, SRR957291, and SRR957297. For selected genes (i.e., MCP and DNA-packaging ATPase sequences), we confirmed their LGT directions with the use of phylogenetic tree reconstruction.

### Proteome analysis of purified medusavirus.

Following virus collection, medusavirus was further purified using 10% to 60% sucrose density gradient centrifugation (2,300 × *g*, 86 min, 4°C). A white-colored virus fraction with a sucrose gradient of approximately 10% to 20% was resuspended in PBS and washed twice with PBS with subsequent centrifugation (8,000 × *g*, 35 min, 4°C). Medusavirus particles were resuspended in PBS containing 0.5% SDS and protease inhibitor cocktail (product no. 25955-11; Nacalai Tesque), and incubated for 1 h at 65°C. Samples were subjected to trichloroacetic acid (TCA) precipitation followed by resuspension in 250 mM Tris-HCl (pH 8.5) containing 2 mM EDTA, and protein was quantified by the bicinchoninic acid (BCA) method. Proteins were reduced for 2 h at 37°C with 0.67 M dithiothreitol (DTT) in 250 mM Tris-HCl (pH 8.5) containing 2 mM EDTA, subsequently alkylated with 1.4 M iodoacetamide in 250 mM Tris-HCl (pH 8.5) containing 2 mM EDTA for 30 min at room temperature, and treated with trypsin for 20 h at 37°C. After desalination and concentration, the treated proteins were subjected to liquid chromatography-tandem mass spectrometry (LC-MS/MS) analysis using the East-nLC 1200 system (Thermo Fisher Scientific Inc., USA) and a Q Exactive Plus spectrometer (Thermo Fisher Scientific Inc., USA). All spectra data were then subjected to NCBI homology search using the Mascot server (http://www.matrixscience.com/server.html).

### Data availability.

The electron microscopy (EM) density maps of the DNA-filled and empty medusavirus particles reported in this paper have been deposited in the EMDatabank **(** http://emdatabank.org) under accession no. EMD-9619 and EMD-9620, respectively. The genome sequence of medusavirus has been submitted to DDBJ under accession no. AP018495. The mass spectrometry proteomics data have been deposited to the Japan ProteOme STandard Repository (jPOSTrepo) under jPOST identifier JPST000467 (https://repository.jpostdb.org/entry/JPST000467) and PXID identifier PXD010830 (http://proteomecentral.proteomexchange.org/cgi/GetDataset?ID=PXD010830).

## Supplementary Material

Supplemental file 1
