# Supplementary material for: Medusavirus, a Novel Large DNA Virus Discovered from Hot Spring Water
Source: J Virol. 2019 Apr 3;93(8):e02130-18. doi: 10.1128/JVI.02130-18 (PMC6450098; doi:10.1128/JVI.02130-18)
Supplement: Supplemental file 1 [file JVI.02130-18-s0001.pdf]

SUPPLEMENTAL MATERIAL

Medusavirus, a Novel Large DNA Virus Discovered from Hot Spring Water

Genki Yoshikawa[1], Romain Blanc-Mathieu[1], Chihong Song[2], Yoko Kayama[2], Tomohiro Mochizuki[3], Kazuyoshi Murata[2,*], Hiroyuki Ogata[1,*], Masaharu Takemura[4,*]

[1] Institute for Chemical Research, Kyoto University, Gokasho, Uji, Kyoto, 611-0011, Japan

[2] National Institute for Physiological Sciences, 38 Nishigonaka Myodaiji, Okazaki, Aichi, 444-8585, Japan

[3] Earth-Life Science Institute, Tokyo Institute of Technology, 2-12-1 Ookayama, Meguro, Tokyo, 152-8550, Japan

[4] Faculty of Science, Tokyo University of Science, 1-3 Kagurazaka, Shinjuku, Tokyo, 162-8601, Japan

[*] Corresponding authors

SUPPLEMENTAL MATERIAL file contains:

Supplemental TABLE S1

Table S1. Medusavirus genes.

ORF no.	Start	End	Strand	Length (aa)	Annotation	No. of introns	BLAST best hit Uniref90 gene ID	Organism group	Organism	E-value	% identity
1	261	647	-	128	hypothetical protein						
2	706	1308	+	200	putative HD hydrolase		A0A1X6WFU3	Viruses	Pacmanvirus A23	1.3E-48	45.6
3	1356	1712	+	118	hypothetical protein						
4	1756	2223	+	155	hypothetical protein						
5	2227	3456	-	409	Rho termination factor N-terminal domain-containing protein		L8GSA9	Eukaryota	Acanthamoeba castellanii str. Neff	6.5E-85	39.0
6	3545	4138	+	197	F-box domain-containing protein		A0A0B5J9H9	Viruses	Pandoravirus inopinatum	1.2E-09	34.8
7	4162	5202	+	346	hypothetical protein						
8	5189	5725	+	178	hypothetical protein		A0A0M4JK20	Viruses	Mollivirus sibericum	1.2E-07	21.3
9	5850	6590	+	246	hypothetical protein		Q5UNY7	Viruses	Mimiviridae	3.6E-54	38.3
10	6607	6804	-	65	hypothetical protein						
11	6811	7158	-	115	hypothetical protein						
12	7336	12327	+	1663	Ser/Thr protein kinase		W5S4J6	Viruses	Pithovirus sibericum	0.0E+00	47.2
13	12348	12545	-	65	hypothetical protein						
14	12579	12881	-	100	hypothetical protein						
15	12973	13437	+	154	hypothetical protein		A0A0M4JT52	Viruses	Mollivirus sibericum	9.6E-74	70.7
16	13446	14270	-	274	hypothetical protein						
17	14533	14832	-	99	hypothetical protein						
18	14841	15314	-	157	hypothetical protein						
19	15657	16043	-	128	DUF2493 domain-containing protein		U2CAU6	Bacteria	Capnocytophaga sp. oral taxon 863 str. F0517	5.3E-27	47.2
20	16175	16405	+	76	hypothetical protein						
21	16569	17063	-	164	hypothetical protein						
22	17163	17738	-	191	hypothetical protein						
23	17782	18426	-	214	hypothetical protein		L8GUY6	Eukaryota	Acanthamoeba castellanii str. Neff	1.2E-29	44.2
24	18565	20628	+	687	putative glycosyltransferase		A0A0M4JK29	Viruses	Mollivirus sibericum	0.0E+00	46.9
25	20625	20909	-	94	hypothetical protein						
26	21076	21273	+	65	hypothetical protein						

27	21270	23357	-	695	DEDDy 3'-5' exonuclease		A0A0M5KJQ5	Viruses	Mollivirus sibericum	4.1E-128	38.0
28	23423	24094	-	223	hypothetical protein						
29	24191	24463	-	90	hypothetical protein						
30	24521	24901	-	126	hypothetical protein						
31	24973	25290	+	105	hypothetical protein						
32	25381	25641	+	86	hypothetical protein						
33	25657	26064	-	135	putative GIY-YIG endonuclease		A0A0M4JAS0	Viruses	Mollivirus sibericum	4.3E-44	55.1
34	26137	26352	+	71	hypothetical protein						
35	26509	26898	-	129	hypothetical protein		F2U6R5	Eukaryota	Salpingoeca rosetta (strain ATCC 50818 / BSB-021)	2.8E-05	30.8
36	26910	27230	-	106	hypothetical protein						
37	27337	28644	+	435	VV A32-like virion packaging ATPase		A0A1Z8URQ2	Bacteria	Candidatus Puniceispirillum sp. TMED52	1.3E-15	26.9
38	28654	29427	-	257	hypothetical protein						
39	29455	29637	+	60	hypothetical protein						
40	29702	31003	-	433	nucleotide sugar dehydrogenase		UPI0009A4E484	Bacteria	Zoogloea sp. LCSB751	0.0E+00	85.3
41	31109	31741	+	210	thymidylate kinase		A0A2D6TMM9	Archaea	Candidatus Pacearchaeota archaeon	2.9E-39	37.8
42	31745	31969	+	74	hypothetical protein						
43	31966	32400	-	144	hypothetical protein						
44	32468	32767	-	99	hypothetical protein		A0A291ATT6	Viruses	Pandoravirus salinus	5.3E-19	47.6
45	32888	33274	+	128	hypothetical protein						
46	33281	33697	-	138	hypothetical protein						
47	33849	35162	+	437	MORN repeat-containing protein		A0A1M7RUL8	Bacteria	Oceanicella actignis	1.1E-14	33.7
48	35176	35631	-	151	hypothetical protein		L8GGG3	Eukaryota	Acanthamoeba castellanii str. Neff	8.2E-50	51.0
49	35712	36080	+	122	hypothetical protein						
50	36142	36426	+	94	hypothetical protein						
51	36418	36693	-	91	hypothetical protein						
52	36865	37080	+	71	hypothetical protein						
53	37177	38742	+	521	hypothetical protein						
54	38750	39043	-	97	hypothetical protein						

55	39091	39651	-	186	hypothetical protein						
56	39795	41003	-	402	hypothetical protein						
57	41149	41553	+	134	hypothetical protein						
58	41572	42399	-	275	hypothetical protein						
59	42587	43045	+	152	hypothetical protein						
60	43123	43734	+	203	hypothetical protein						
61	43781	44341	-	186	histone H2B		L8H4F8	Eukaryota	Acanthamoeba castellanii str. Neff	9.8E-06	36.4
62	44503	45198	-	231	hypothetical protein		L8GS30	Eukaryota	Acanthamoeba castellanii str. Neff	8.6E-45	51.9
63	45430	45993	-	187	hypothetical protein						
64	46069	47076	-	335	hypothetical protein		A0A2E4HL59	Bacteria	Nitrospinae bacterium	3.5E-04	22.3
65	47220	47393	+	57	hypothetical protein						
66	47399	48022	-	207	hypothetical protein						
67	48192	49604	-	470	hypothetical protein						
68	49763	52639	+	958	hypothetical protein						
69	52668	52910	-	80	hypothetical protein						
70	52925	54385	+	486	hypothetical protein						
71	54414	55658	+	414	YqaJ viral recombinase		M4QS94		root	2.7E-44	47.3
72	55670	56290	-	206	hypothetical protein						
73	56332	56481	-	49	hypothetical protein						
74	56480	58255	+	591	hypothetical protein		L8GRL8	Eukaryota	Acanthamoeba castellanii str. Neff	9.3E-39	40.8
75	58287	58970	-	227	DNA methyltransferase		A0A1V3K7B5	Bacteria	Rodentibacter pneumotropicus	1.3E-48	47.5
76	59043	59558	-	171	hypothetical protein						
77	59590	60147	-	185	hypothetical protein		S4VZD6	Viruses	Pandoravirus dulcis	7.7E-18	33.5
78	60215	60460	-	81	hypothetical protein						
79	60495	61337	-	280	F-box domain-containing protein						
80	61474	62055	+	193	hypothetical protein						
81	62139	63323	+	394	hypothetical protein						
82	63320	64045	-	241	hypothetical protein						

83	64116	64580	+	154	hypothetical protein						
84	64604	64918	-	104	hypothetical protein						
85	64979	65224	-	81	hypothetical protein						
86	65325	65621	+	98	hypothetical protein		L8GZP6	Eukaryota	Acanthamoeba castellanii str. Neff	1.2E-16	45.3
87	65622	66584	-	320	Ser/Thr protein kinase		A0A1S3GM49	Eukaryota	Dipodomys ordii	2.4E-22	30.8
88	66651	67034	+	127	hypothetical protein						
89	67101	67337	+	78	hypothetical protein						
90	67418	68491	+	357	hypothetical protein						
91	68452	68679	+	75	hypothetical protein						
92	68691	69461	-	256	zf-PARP domain-containing protein						
93	69531	70853	-	440	DUF4804 domain-containing protein		A0A0U5JC34	Bacteria	Candidatus Protochlamydia	5.1E-69	35.0
94	70928	71827	-	299	hypothetical protein						
95	71967	72587	+	206	hypothetical protein						
96	72637	72978	+	113	hypothetical protein						
97	72989	73768	-	259	hypothetical protein						
98	73917	74927	+	336	hypothetical protein						
99	75015	76262	+	415	hypothetical protein						
100	76257	77132	-	291	hypothetical protein						
101	77139	77276	-	45	hypothetical protein						
102	77308	77862	-	184	hypothetical protein						
103	78016	78825	-	269	hypothetical protein		L8GWL9	Eukaryota	Acanthamoeba castellanii str. Neff	7.8E-78	51.0
104	78910	79113	+	67	hypothetical protein						
105	79169	79402	+	77	hypothetical protein		A0A1X6WFR4	Viruses	Pacmanvirus A23	3.8E-14	46.8
106	79719	80234	-	171	linker histone H1		L8GT86	Eukaryota	Acanthamoeba castellanii str. Neff	2.0E-27	42.8
107	80359	81555	-	398	hypothetical protein						
108	81872	83044	-	390	hypothetical protein						
109	83118	84104	-	328	F-box domain-containing protein		L8GRP9	Eukaryota	Acanthamoeba castellanii str. Neff	1.3E-17	39.7
110	84164	85099	-	311	hypothetical protein		UPI0006B61C07	Archaea	Halolamina sediminis	2.7E-09	27.3

111	85273	86046	-	257	hypothetical protein		L8GIN3	Eukaryota	Acanthamoeba castellanii str. Neff	1.2E-70	55.6
112	86112	86228	-	38	hypothetical protein						
113	86231	87043	-	270	hypothetical protein		L8GWL9	Eukaryota	Acanthamoeba castellanii str. Neff	6.9E-54	41.1
114	87104	87394	+	96	hypothetical protein						
115	87405	87968	-	187	hypothetical protein						
116	88103	88804	+	233	Rho termination factor N-terminal domain-containing protein		A0A2E6F125	Bacteria	Flavobacteriaceae	3.3E-04	42.9
117	88811	89650	-	279	hypothetical protein						
118	89773	90477	+	234	hypothetical protein						
119	90445	90711	-	88	hypothetical protein						
120	90790	91956	+	388	hypothetical protein		F2WL63		Viruses	4.5E-88	42.8
121	91996	92235	+	79	hypothetical protein						
122	92250	93002	+	250	hypothetical protein		L8GWL9	Eukaryota	Acanthamoeba castellanii str. Neff	7.0E-35	42.1
123	93044	93838	+	264	PKD domain-containing protein		L8GXT2	Eukaryota	Acanthamoeba castellanii str. Neff	3.6E-12	30.0
124	93852	95024	-	390	hypothetical protein						
125	95191	96474	+	427	poly A polymerase regulatory subunit		A0A0D2UAZ4	Eukaryota	Capsaspora owczarzaki (strain ATCC 30864)	3.6E-94	54.3
126	96493	96675	-	60	hypothetical protein						
127	96739	97959	-	406	hypothetical protein						
128	98097	98483	+	128	hypothetical protein						
129	98471	98644	-	57	hypothetical protein		A0A291ATW7	Viruses	Pandoravirus dulcis	4.6E-20	75.0
130	98650	98889	-	79	hypothetical protein						
131	98919	99362	-	147	hypothetical protein						
132	99443	99748	-	101	hypothetical protein		A0A0M4JJL7	Viruses	Mollivirus sibericum	2.7E-06	28.9
133	99829	100032	+	67	hypothetical protein						
134	100241	100606	+	121	hypothetical protein		L8GM37	Eukaryota	Acanthamoeba castellanii str. Neff	4.4E-10	35.0
135	100658	101092	+	144	hypothetical protein						
136	101102	101878	-	258	hypothetical protein						
137	101882	102694	-	270	hypothetical protein						
138	102712	103575	+	287	PIN domain-containing protein		L8GYG2	Eukaryota	Acanthamoeba castellanii str. Neff	1.8E-75	44.5

139	103637	104431	+	264	BTB/POZ domain-containing protein		L8HHL4	Eukaryota	Acanthamoeba castellanii str. Neff	7.1E-33	38.4
140	104446	105576	-	376	hypothetical protein		L8GYB5	Eukaryota	Acanthamoeba castellanii str. Neff	1.1E-37	36.5
141	105645	106766	-	373	hypothetical protein		L8GYB5	Eukaryota	Acanthamoeba castellanii str. Neff	1.1E-33	35.7
142	106860	107138	-	92	hypothetical protein						
143	107292	107624	+	110	hypothetical protein						
144	107631	108170	-	179	hypothetical protein						
145	108287	110536	+	749	Ser/Thr protein kinase		UPI000BE16838	Eukaryota	Gamasina	3.1E-05	29.9
146	110545	110757	-	70	hypothetical protein						
147	110840	111397	+	185	hypothetical protein						
148	111394	112410	-	338	Ser/Thr protein kinase		A0A0M4K4H8	Viruses	Mollivirus sibericum	1.6E-103	52.3
149	112930	113241	+	103	high mobility group protein		A0A0H5R7U8	Eukaryota	Spongospora subterranea	3.9E-18	50.0
150	113271	113918	-	215	hypothetical protein						
151	113967	114152	-	61	hypothetical protein						
152	114203	114667	+	154	hypothetical protein						
153	114795	115085	-	96	hypothetical protein						
154	115138	115518	-	126	hypothetical protein		L8H9H2	Eukaryota	Acanthamoeba castellanii str. Neff	3.5E-27	61.3
155	115589	115912	+	107	hypothetical protein						
156	115921	117018	-	365	F-box domain-containing protein		L8GNI3	Eukaryota	Acanthamoeba castellanii str. Neff	4.3E-15	62.9
157	117091	118497	-	468	ankyrin repeat protein		S4VVT0	Viruses	Pandoravirus salinus	6.5E-147	51.8
158	118540	120177	-	545	hypothetical protein		A0A182QRX9	Eukaryota	Anopheles farauti	7.4E-08	29.1
159	120268	120816	+	182	hypothetical protein						
160	120807	121019	-	70	hypothetical protein						
161	121598	121882	-	94	hypothetical protein						
162	122571	122837	+	88	hypothetical protein		A0A0M3SGV6	Viruses	Mollivirus sibericum	4.5E-04	36.5
163	122824	123105	-	93	hypothetical protein						
164	123131	123304	-	57	hypothetical protein						
165	123311	123421	-	36	hypothetical protein						
166	123431	123883	-	150	hypothetical protein		D2XA65	Viruses	Marseilleviridae	9.6E-18	34.7

167	124002	124802	-	266	hypothetical protein		A0A1Q1PNN5	Viruses	unclassified Marseilleviridae	5.8E-58	44.1
168	125236	125373	-	45	hypothetical protein						
169	126121	126795	-	224	zf-PARP domain-containing protein		A0A0C9XC42	Eukaryota	Laccaria amethystina LaAM-08-1	7.5E-08	41.9
170	127111	127458	-	115	hypothetical protein						
171	127491	127859	+	122	hypothetical protein						
172	127881	128294	-	137	hypothetical protein						
173	128377	128619	-	80	hypothetical protein						
174	128714	128911	+	65	hypothetical protein						
175	128942	129490	-	182	hypothetical protein		F2WL38	Viruses	Lausannevirus	2.5E-18	31.3
176	129683	130951	+	422	putative HNH endonuclease		Q98528	Viruses	Chlorovirus	1.1E-21	37.7
177	131116	132632	-	481	major capsid protein	1	L8GRF1	Eukaryota	Acanthamoeba castellanii str. Neff	1.4E-174	75.7
178	132883	133665	+	260	hypothetical protein		L8GP88	Eukaryota	Acanthamoeba castellanii str. Neff	4.2E-23	35.3
179	133672	141015	-	2376	hypothetical protein	1	L8GMS7	Eukaryota	Acanthamoeba castellanii str. Neff	1.0E-44	37.6
180	141117	141824	-	235	hypothetical protein		X0STG9	metageno mes	marine sediment metagenome	5.0E-11	31.5
181	141921	142598	+	225	hypothetical protein						
182	142603	143106	-	167	glutaredoxin		UPI0001778D1C	Eukaryota	Ochotona princeps	2.1E-05	39.7
183	143169	143882	+	237	hypothetical protein						
184	143839	144144	-	101	hypothetical protein						
185	144243	144635	+	130	hypothetical protein						
186	144589	145785	-	398	hypothetical protein		L8GPY3	Eukaryota	Acanthamoeba castellanii str. Neff	1.8E-36	30.9
187	145883	146245	+	120	hypothetical protein						
188	146253	149245	+	962	hypothetical protein	1	L8GRE8	Eukaryota	Acanthamoeba castellanii str. Neff	7.0E-74	47.4
189	149306	149695	+	129	hypothetical protein						
190	149735	152296	+	853	hypothetical protein		L8GSA9	Eukaryota	Acanthamoeba castellanii str. Neff	3.5E-38	32.1
191	152304	152561	+	85	hypothetical protein						
192	152616	153632	+	338	putative holliday junction resolvase						
193	153639	154238	-	199	hypothetical protein						
194	154339	155937	+	532	hypothetical protein						

195	155859	156926	-	355	hypothetical protein		A0A0B4CHN1	Bacteria	Brevundimonas nasdae	1.7E-05	33.7
196	157265	157630	+	121	putative late transcription factor VLTF-3		A0A2E3ZXZ5	Bacteria	Candidatus Marinimicrobia bacterium	5.4E-19	39.3
197	158067	159365	+	432	hypothetical protein		L8GY54	Eukaryota	Acanthamoeba castellanii str. Neff	7.3E-47	39.3
198	159437	159877	+	146	hypothetical protein						
199	159954	160934	+	326	hypothetical protein		L8GTM8	Eukaryota	Acanthamoeba castellanii str. Neff	1.1E-08	43.2
200	160938	162638	-	566	hypothetical protein		L8GL54	Eukaryota	Acanthamoeba castellanii str. Neff	1.5E-10	28.6
201	162804	163868	+	354	hypothetical protein						
202	163955	164278	+	107	hypothetical protein		S4VYB1	Viruses	Pandoravirus salinus	2.5E-24	50.0
203	164275	164853	-	192	molybdenum cofactor carrier		L8H0L9	Eukaryota	Acanthamoeba castellanii str. Neff	9.9E-43	78.9
204	164927	165634	+	235	BTB/POZ domain-containing protein		M0QSF8	Eukaryota	Acanthamoeba castellanii str. Neff	1.1E-21	41.2
205	165701	166147	+	148	hypothetical protein						
206	166144	167109	-	321	hypothetical protein		A0A0B5JBD3	Viruses	Pandoravirus inopinatum	4.2E-132	58.6
207	167171	167614	-	147	hypothetical protein						
208	167687	168364	-	225	hypothetical protein						
209	168482	168943	-	153	dual specificity phosphatase		S4VXU9	Viruses	Pandoravirus dulcis	1.9E-40	53.5
210	169012	169938	-	308	hypothetical protein		L8GF30	Eukaryota	Acanthamoeba castellanii str. Neff	2.8E-58	69.2
211	169995	170534	-	179	macro domain-containing protein		UPI000BAEC65D	Eukaryota	Crassostrea virginica	1.3E-35	42.8
212	170648	170845	+	65	hypothetical protein						
213	170929	171270	+	113	hypothetical protein		L8GEF1	Eukaryota	Acanthamoeba castellanii str. Neff	1.2E-43	67.9
214	171309	172310	+	333	hypothetical protein		L8GH06	Eukaryota	Acanthamoeba castellanii str. Neff	2.2E-21	27.7
215	172322	172906	-	194	serine hydrolase		S4VV52	Viruses	Pandoravirus salinus	1.1E-76	60.1
216	173005	173337	+	110	hypothetical protein						
217	173343	173654	-	103	hypothetical protein						
218	173722	174030	+	102	hypothetical protein						
219	174040	175419	-	459	beta-galactosidase		A0A0B5JD41	Viruses	Pandoravirus inopinatum	0.0E+00	62.7
220	175464	176168	-	234	G2/mitotic-specific cyclin		L8GRU0	Eukaryota	Acanthamoeba castellanii str. Neff	2.8E-32	34.5
221	176241	176702	-	153	hypothetical protein						
222	176846	177424	+	192	hypothetical protein						

223	177856	178035	-	59	hypothetical protein						
224	178226	179128	-	300	ribonuclease HII		L8GDC2	Eukaryota	Acanthamoeba castellanii str. Neff	5.5E-79	55.0
225	179194	180261	+	355	hypothetical protein		L8GYB5	Eukaryota	Acanthamoeba castellanii str. Neff	4.1E-50	36.9
226	180274	181422	-	382	putative membrane protein		L8GVP3	Eukaryota	Acanthamoeba castellanii str. Neff	2.2E-128	59.1
227	181505	181762	-	85	hypothetical protein						
228	181876	182484	+	202	hypothetical protein						
229	182481	183516	-	322	putative VV A32-like packaging ATPase	1	L8GKI6	Eukaryota	Acanthamoeba castellanii str. Neff	1.5E-57	63.5
230	183756	184913	+	385	hypothetical protein		M1GXL2	Viruses	Acanthocystis turfacea Chlorella virus Canal-1	7.8E-49	37.2
231	184908	185153	-	81	hypothetical protein						
232	185262	186266	-	334	hypothetical protein		L8GKI6	Eukaryota	Acanthamoeba castellanii str. Neff	1.1E-13	32.9
233	186344	187006	+	220	hypothetical protein						
234	187065	187583	+	172	hypothetical protein						
235	187538	188095	-	185	hypothetical protein						
236	188145	190112	-	655	hypothetical protein		L8GKD3	Eukaryota	Acanthamoeba castellanii str. Neff	0.0E+00	48.8
237	190176	190568	-	130	hypothetical protein						
238	190639	191022	-	127	hypothetical protein						
239	191089	191502	-	137	hypothetical protein						
240	191567	191782	-	71	hypothetical protein						
241	191842	192255	-	137	hypothetical protein						
242	192314	193582	-	422	hypothetical protein						
243	193795	194658	+	287	hypothetical protein						
244	194699	195508	+	269	putative tRNA-His guanylyltransferase		A0A1Y2WX95	Eukaryota	Daldinia sp. EC12	3.4E-04	25.0
245	195531	196253	+	240	putative RING finger protein		L8HI87	Eukaryota	Acanthamoeba castellanii str. Neff	2.9E-05	47.4
246	196325	196537	+	70	hypothetical protein						
247	196600	196842	+	80	hypothetical protein						
248	196866	197423	-	185	hypothetical protein						
249	197448	197957	+	169	hypothetical protein						
250	197987	198688	+	233	hypothetical protein						

251	198904	199200	-	98	hypothetical protein						
252	199247	199462	-	71	hypothetical protein						
253	199527	200072	-	181	hypothetical protein						
254	200186	200464	-	92	histone H4						
255	200585	201076	-	163	histone H3		P08898		cellular organisms	1.4E-21	46.3
256	201105	202208	-	367	hypothetical protein		L8GSA9	Eukaryota	Acanthamoeba castellanii str. Neff	1.3E-05	25.2
257	202268	202468	+	66	hypothetical protein						
258	202524	204506	+	660	hypothetical protein						
259	204511	205017	-	168	hypothetical protein						
260	205171	205896	+	241	hypothetical protein						
261	206110	206643	-	177	hypothetical protein		L8GVH1	Eukaryota	Acanthamoeba castellanii str. Neff	6.9E-27	51.7
262	206857	210141	+	1094	hypothetical protein		L8GSA9	Eukaryota	Acanthamoeba castellanii str. Neff	1.9E-27	28.7
263	210178	215310	-	1710	Ser/Thr protein kinase		S4W2F5	Viruses	Pandoravirus salinus	0.0E+00	34.8
264	215519	216562	+	347	thymidylate synthase		A0A1X2HS00	Eukaryota	Syncephalastrum racemosum	2.5E-93	43.5
265	216602	217042	+	146	hypothetical protein		L8HJD0	Eukaryota	Acanthamoeba castellanii str. Neff	2.6E-09	35.8
266	217091	217462	+	123	hypothetical protein						
267	217459	218706	-	415	exonuclease domain-containing protein						
268	218758	220086	-	442	hypothetical protein		Q8QNH4		root	1.9E-09	26.3
269	220178	220798	+	206	AAA domain-containing protein		L8GI16	Eukaryota	Acanthamoeba castellanii str. Neff	1.2E-60	52.2
270	220828	220995	+	55	hypothetical protein						
271	221028	221861	+	277	hypothetical protein						
272	221946	223100	-	384	hypothetical protein						
273	223197	223649	+	150	hypothetical protein						
274	223692	224153	+	153	hypothetical protein						
275	224237	224836	+	199	hypothetical protein						
276	224881	225351	+	156	hypothetical protein						
277	225361	225903	-	180	hypothetical protein						
278	225931	226149	-	72	hypothetical protein						

279	226273	228847	+	637	ribonucleotide reductase large subunit	1	L8HMN7	Eukaryota	Acanthamoeba castellanii str. Neff	0.0E+00	71.7
280	228877	229110	-	77	hypothetical protein						
281	229112	230176	-	354	hypothetical protein		L8GID9	Eukaryota	Acanthamoeba castellanii str. Neff	9.3E-148	55.2
282	230221	231039	-	272	class 3 lipase		L8GV53	Eukaryota	Acanthamoeba castellanii str. Neff	9.2E-42	39.3
283	231157	231870	+	237	hypothetical protein						
284	231934	233055	+	373	hypothetical protein						
285	233062	233484	-	140	hypothetical protein		L8H0K1	Eukaryota	Acanthamoeba castellanii str. Neff	5.7E-11	71.1
286	233516	234820	-	434	hypothetical protein		B5LJI0	Viruses	Mycobacterium phage Myrna	1.6E-10	34.6
287	234907	236136	-	409	ribonucleotide reductase small subunit		S4VT34	Viruses	Pandoravirus dulcis	7.4E-155	61.8
288	236257	236631	+	124	hypothetical protein						
289	236622	237656	-	344	hypothetical protein						
290	237704	238834	-	376	hypothetical protein		L8GXS6	Eukaryota	Acanthamoeba castellanii str. Neff	1.0E-50	38.0
291	238918	239223	-	101	hypothetical protein						
292	239361	239915	+	184	hypothetical protein						
293	240189	240377	+	62	hypothetical protein						
294	240408	240671	-	87	hypothetical protein						
295	240769	241158	+	129	hypothetical protein						
296	241177	241644	-	155	hypothetical protein						
297	241726	242271	-	181	hypothetical protein		S4VNY1	Viruses	unclassified dsDNA viruses	2.4E-89	70.7
298	242306	243199	-	297	dihydrofolate reductase		S4VY84	Viruses	Pandoravirus salinus	3.0E-11	35.8
299	243244	243678	-	144	hypothetical protein		L8GPM9	Eukaryota	Acanthamoeba castellanii str. Neff	1.4E-07	36.0
300	243769	244596	+	275	BTB/POZ domain-containing protein		A0A2G5UXP4	Eukaryota	Caenorhabditis nigoni	2.0E-05	31.4
301	244670	245107	+	145	hypothetical protein						
302	245223	245858	+	211	VV A32-like virion packaging ATPase		A0A0B5J5F3	Viruses	Pandoravirus inopinatum	2.0E-14	29.9
303	245882	246844	-	320	hypothetical protein		L1J4A4	Eukaryota	Guillardia theta CCMP2712	4.9E-07	26.8
304	247228	247845	+	205	hypothetical protein						
305	247881	248066	-	61	hypothetical protein						
306	248180	249769	-	529	hypothetical protein		L8GNI6	Eukaryota	Acanthamoeba castellanii str. Neff	7.4E-52	44.4

307	249936	250784	+	282	putative proliferating cell nuclear antigen						
308	250818	253151	-	777	hypothetical protein		L8GNI6	Eukaryota	Acanthamoeba castellanii str. Neff	0.0E+00	56.8
309	253170	254117	-	315	F-box domain-containing protein		L8GRP9	Eukaryota	Acanthamoeba castellanii str. Neff	5.1E-31	56.0
310	254159	255499	-	446	hypothetical protein		L8GY54	Eukaryota	Acanthamoeba castellanii str. Neff	1.6E-97	62.3
311	255589	255747	+	52	hypothetical protein						
312	255841	256656	+	271	DNA-3-methyladenine glycosylase		A0A0M4JB55	Viruses	Mollivirus sibericum	2.3E-56	45.8
313	256662	257255	-	197	hypothetical protein						
314	257321	257719	-	132	hypothetical protein						
315	257798	258445	+	215	hypothetical protein						
316	258532	259086	+	184	deoxycytidylate deaminase		A0A0M4K765	Viruses	Mollivirus sibericum	6.3E-71	60.2
317	259250	260023	-	257	hypothetical protein						
318	260170	260871	+	233	histone H2A		H9JMY0	Eukaryota	Bombyx mori	1.1E-09	41.2
319	261013	263145	+	710	PIF1-like helicase		W7HWQ6	Eukaryota	Drechslerella stenobrocha 248	3.6E-50	30.6
320	263178	263393	+	71	hypothetical protein						
321	263414	265012	-	532	Rho termination factor N-terminal domain-containing protein		L8GSA9	Eukaryota	Acanthamoeba castellanii str. Neff	1.2E-34	31.2
322	265143	265331	+	62	hypothetical protein						
323	265297	265653	+	118	hypothetical protein						
324	265662	266813	-	383	Ser/Thr protein kinase		Q8H1P2	Eukaryota	Soja	1.7E-16	31.0
325	267124	268866	+	580	hypothetical protein						
326	268909	270204	-	431	hypothetical protein						
327	270397	271188	+	263	hypothetical protein						
328	271292	271888	+	198	hypothetical protein						
329	271951	272709	+	252	putative myristoylated membrane protein		L8GWW3	Eukaryota	Acanthamoeba castellanii str. Neff	4.9E-55	49.8
330	272764	273573	+	269	DUF4326 domain-containing protein		L8GMA6	Eukaryota	Acanthamoeba castellanii str. Neff	2.1E-50	38.6
331	273624	275816	+	730	hypothetical protein		UPI000769C8AA	Bacteria	Aliivibrio wodanis	1.1E-09	26.3
332	275852	276508	+	218	hypothetical protein						
333	276505	277167	+	220	hypothetical protein						
334	277238	278137	+	299	F-box domain-containing protein						

335	278248	278436	+	62	hypothetical protein						
336	278524	278784	+	86	hypothetical protein						
337	278826	280973	+	715	FG-GAP repeat-containing protein		A0A1X6WEM0	Viruses	Pacmanvirus A23	9.6E-15	30.4
338	281033	281677	+	214	hypothetical protein						
339	281736	282395	+	219	hypothetical protein						
340	282471	283505	+	344	nucleoside diphosphate kinase		L8GWD2	Eukaryota	Acanthamoeba castellanii str. Neff	1.7E-53	36.6
341	283521	283922	-	133	PAN domain-containing protein		L8GYA1	Eukaryota	Acanthamoeba castellanii str. Neff	5.0E-20	39.4
342	283980	284345	-	121	PAN domain-containing protein		L8GYA1	Eukaryota	Acanthamoeba castellanii str. Neff	1.1E-21	42.5
343	284459	284581	+	40	hypothetical protein						
344	284604	285347	-	247	hypothetical protein						
345	285513	285779	+	88	hypothetical protein						
346	285853	286659	-	268	proliferating cell nuclear antigen		A0A2B4RZH5	Eukaryota	Stylophora pistillata	1.6E-34	31.6
347	286720	287256	-	178	hypothetical protein						
348	287401	287895	-	164	deoxyuridine triphosphatase		K7PC95	Viruses	Cyprinid herpesvirus 1	6.8E-36	50.0
349	287882	288421	-	179	thioredoxin		L8GZB6	Eukaryota	Acanthamoeba castellanii str. Neff	2.4E-09	42.3
350	288476	288640	-	54	hypothetical protein						
351	288700	289377	-	225	hypothetical protein		L8H9G2	Eukaryota	Acanthamoeba castellanii str. Neff	2.6E-31	47.4
352	289485	289892	+	135	hypothetical protein						
353	290071	290625	+	184	hypothetical protein						
354	290661	291173	+	170	hypothetical protein		L8HBP7	Eukaryota	Acanthamoeba castellanii str. Neff	1.3E-21	37.3
355	291247	291579	+	110	hypothetical protein						
356	291700	292281	-	173	GTP binding nuclear protein Ran	1	L8GL91	Eukaryota	Acanthamoeba castellanii str. Neff	4.8E-42	47.7
357	292283	293038	-	251	hypothetical protein		L8HAS6	Eukaryota	Acanthamoeba castellanii str. Neff	5.8E-14	36.9
358	293120	293410	+	96	hypothetical protein						
359	293473	294027	+	184	hypothetical protein						
360	294040	294438	+	132	hypothetical protein						
361	294480	295979	+	499	Rho termination factor N-terminal domain-containing protein		L8GZ43	Eukaryota	Acanthamoeba castellanii str. Neff	1.1E-05	39.8
362	295991	296482	-	163	hypothetical protein						

363	296590	297252	-	220	ribonuclease H		W2VFG2	Bacteria	Lachnoanaerobaculum	2.0E-08	26.5
364	297321	297647	+	108	hypothetical protein						
365	297671	299545	+	624	replication factor C large subunit		L8HIF4	Eukaryota	Acanthamoeba castellanii str. Neff	0.0E+00	44.6
366	299552	299971	-	139	hypothetical protein						
367	300089	300310	+	73	hypothetical protein						
368	300307	300948	-	213	hypothetical protein		M1I381	Viruses	Chlorovirus	4.0E-14	25.7
369	301075	301836	-	253	hypothetical protein		L8GVN3	Eukaryota	Acanthamoeba castellanii str. Neff	3.4E-10	24.6
370	301924	302076	+	50	hypothetical protein						
371	302132	302272	-	46	hypothetical protein						
372	302458	303393	-	311	WD40 domain-containing protein		L8H808	Eukaryota	Acanthamoeba castellanii str. Neff	6.9E-31	27.6
373	303447	303662	-	71	hypothetical protein						
374	303711	303830	+	39	hypothetical protein						
375	303827	306421	-	864	DNA helicase		L8HBP1	Eukaryota	Acanthamoeba castellanii str. Neff	4.3E-165	50.2
376	306591	307082	+	163	hypothetical protein						
377	307090	307275	-	61	hypothetical protein						
378	307411	307899	+	162	hypothetical protein						
379	307935	308447	-	170	hypothetical protein		A0A2D5VP24	Bacteria	Pseudomonas sp.	4.8E-19	36.1
380	308580	308708	+	42	hypothetical protein						
381	308736	309392	-	218	endonuclease V		F2U0C9	Eukaryota	Salpingoeca rosetta (strain ATCC 50818 / BSB-021)	2.2E-83	56.9
382	309385	309888	-	167	hypothetical protein						
383	310142	310396	+	84	hypothetical protein						
384	310416	310517	-	33	hypothetical protein						
385	310838	311179	+	113	hypothetical protein						
386	311264	311722	+	152	hypothetical protein						
387	311685	312212	-	175	hypothetical protein						
388	312276	312947	-	223	UBA-like domain-containing protein		A0A072V2D6	Eukaryota	Medicago truncatula	4.2E-05	43.8
389	313085	313300	+	71	hypothetical protein						
390	313396	314073	+	225	hypothetical protein						

391	314091	315107	-	338	hypothetical protein						
392	315238	315666	+	142	hypothetical protein						
393	315783	316082	+	99	initiation factor eIF1/SUI		L8GHP9	Eukaryota	Acanthamoeba castellanii str. Neff	1.1E-31	49.0
394	316076	317092	-	338	hypothetical protein						
395	317231	317494	+	87	hypothetical protein						
396	317513	317788	-	91	hypothetical protein						
397	317997	318239	+	80	hypothetical protein						
398	318272	319420	+	382	protein phosphatase 2C		A0A0A1TYY5	Eukaryota	Entamoeba invadens	8.3E-18	28.9
399	319428	319862	+	144	hypothetical protein						
400	319863	320705	+	280	hypothetical protein						
401	320965	321513	+	182	hypothetical protein		A0A146JEC3	Viruses	Tokyovirus A1	5.2E-44	44.9
402	321614	321862	+	82	hypothetical protein						
403	321936	322577	+	213	hypothetical protein		L8GM35	Eukaryota	Acanthamoeba castellanii str. Neff	7.8E-05	42.2
404	322602	322835	-	77	hypothetical protein		L8HD82	Eukaryota	Acanthamoeba castellanii str. Neff	1.5E-30	74.6
405	322911	323435	-	174	hypothetical protein		L8HAT1	Eukaryota	Acanthamoeba castellanii str. Neff	4.1E-10	26.0
406	323491	323952	-	153	SET domain-containing protein		I0XTT5	Bacteria	Leptospiraceae	9.0E-15	34.7
407	324039	324302	+	87	hypothetical protein		L8H0J5	Eukaryota	Acanthamoeba castellanii str. Neff	1.3E-10	37.3
408	324310	324567	-	85	hypothetical protein						
409	324573	327206	-	877	putative DNA primase		A0A0P0YMW5	Viruses	Yellowstone lake phycodnavirus 1	1.3E-49	29.4
410	327330	327740	-	136	hypothetical protein						
411	327811	332290	-	1257	DNA polymerase	3	UPI0006251E38	Eukaryota	Athalia rosae	3.1E-131	29.9
412	332436	333383	+	315	Ser/Thr protein kinase		L8H9C4	Eukaryota	Acanthamoeba castellanii str. Neff	3.8E-84	44.4
413	333436	335877	+	813	hypothetical protein		V6T8N0	Eukaryota	Giardia intestinalis	6.1E-04	28.1
414	335874	337169	-	431	proliferating cell nuclear antigen		A0A2E4HLZ5	Bacteria	Nitrospinae bacterium	3.4E-30	28.8
415	337253	337633	-	126	hypothetical protein						
416	337796	338026	-	76	hypothetical protein						
417	338202	338822	+	206	hypothetical protein		I0YQH0	Eukaryota	Coccomyxa subellipsoidea (strain C-169)	4.3E-06	30.0
418	338964	340259	+	431	hypothetical protein		UPI0006797E21	Archaea	Halapricum salinum	3.0E-12	33.1

419	340555	341334	-	259	metacaspase		UPI0004417EA8	Eukaryota	Punctularia strigosozonata (strain HHB-11173)	4.8E-38	38.3
420	341425	342429	-	334	putative VLTF-2 transcription factor		V5LSF6	Viruses	unclassified Coccolithovirus	1.8E-08	31.2
421	342577	344577	-	666	hypothetical protein		L8GKP6	Eukaryota	Acanthamoeba castellanii str. Neff	1.3E-12	35.2
422	344759	345730	+	323	hypothetical protein		A0A1Y1IQV2	Eukaryota	Klebsormidium nitens	2.7E-15	27.8
423	345811	346188	-	125	hypothetical protein		Q8QNI3	Viruses	Ectocarpus siliculosus virus 1 (isolate New Zealand/Kaikoura/1988)	2.2E-14	41.9
424	346304	347104	+	266	BTB/POZ domain-containing protein						
425	347194	347661	+	155	hypothetical protein						
426	347724	348056	+	110	hypothetical protein						
427	348098	348373	+	91	hypothetical protein						
428	348445	348762	+	105	hypothetical protein						
429	348771	351173	-	800	hypothetical protein		L8GLA0	Eukaryota	Acanthamoeba castellanii str. Neff	5.8E-31	38.2
430	351285	352196	-	303	nudix hydrolase		A0A1X7TYR1	Eukaryota	Amphimedon queenslandica	7.1E-25	30.3
431	352260	352559	+	99	hypothetical protein						
432	352657	355797	+	1046	hypothetical protein						
433	355900	359034	+	1044	LYR motif-containing protein						
434	359081	360241	-	386	hypothetical protein						
435	360304	360912	-	202	hypothetical protein						
436	360956	361615	-	219	hypothetical protein						
437	361674	361835	-	53	hypothetical protein		A0A1Y2HA82	Eukaryota	Catenaria anguillulae PL171	5.5E-06	55.9
438	361978	362706	+	242	hypothetical protein		L8GX11	Eukaryota	Acanthamoeba castellanii str. Neff	1.1E-05	50.0
439	362787	363395	+	202	transcription elongation factor S-II		L8GZ47	Eukaryota	Acanthamoeba castellanii str. Neff	9.4E-24	34.1
440	363422	364411	-	329	hypothetical protein						
441	364541	364966	+	141	hypothetical protein						
442	364972	365619	-	215	hypothetical protein						
443	365669	366340	-	223	hypothetical protein						
444	366369	366626	-	85	hypothetical protein						
445	366695	368863	-	722	hypothetical protein		A0A1X6WEM0	Viruses	Pacmanvirus A23	2.0E-15	28.4
446	369097	370167	+	356	hypothetical protein						

447	370189	370845	-	218	hypothetical protein						
448	370977	372467	+	496	mitochondrial chaperone BCS1		S4VVG3	Viruses	Pandoravirus salinus	2.4E-76	34.3
449	372475	373122	-	215	hypothetical protein						
450	373192	373638	+	148	hypothetical protein						
451	373701	374111	+	136	hypothetical protein						
452	374091	374585	-	164	hypothetical protein		L8HDD7	Eukaryota	Acanthamoeba castellanii str. Neff	4.5E-09	37.9
453	374746	375285	-	179	F-box domain-containing protein						
454	375284	375769	+	161	PAN domain-containing protein		L8GX08	Eukaryota	Acanthamoeba castellanii str. Neff	7.1E-30	44.3
455	375782	376504	-	240	zf-MYND domain-containing protain		UPI000440B3FC	Eukaryota	Stereum hirsutum (strain FP-91666)	1.1E-06	50.0
456	376556	376954	-	132	hypothetical protein						
457	377021	377236	-	71	hypothetical protein						
458	377240	378016	-	258	hypothetical protein						
459	378085	378669	-	194	dihydrofolate reductase		UPI0007E7FC81	Eukaryota	Drosophila biarmipes	5.9E-32	39.0
460	378687	379229	+	180	hypothetical protein						
461	379604	380338	+	244	hypothetical protein						
